# Embedding Atomically Dispersed Iron Sites in Nitrogen‐Doped Carbon Frameworks‐Wrapped Silicon Suboxide for Superior Lithium Storage

**DOI:** 10.1002/advs.202206084

**Published:** 2022-12-05

**Authors:** Xiaotian Guo, Hengyue Xu, Wenting Li, Yangyi Liu, Yuxin Shi, Qing Li, Huan Pang

**Affiliations:** ^1^ School of Chemistry and Chemical Engineering Yangzhou University Yangzhou Jiangsu 225009 P. R. China; ^2^ Institute of Biopharmaceutical and Health Engineering Tsinghua Shenzhen International Graduate School Tsinghua University Shenzhen 518055 P. R. China; ^3^ Guangling College Yangzhou University Yangzhou Jiangsu 225009 P. R. China

**Keywords:** atomically dispersed iron sites, catalytic attribute, Li‐ion‐battery anodes, nitrogen‐doped carbon frameworks, silicon suboxide

## Abstract

Silicon suboxide (SiO*_x_*) has attracted widespread interest as Li‐ion battery (LIB) anodes. However, its undesirable electronic conductivity and apparent volume effect during cycling impede its practical applications. Herein, sustainable rice husks (RHs)‐derived SiO_2_ are chosen as a feedstock to design SiO*_x_*/iron–nitrogen co‐doped carbon (Fe–N–C) materials. Using a facile electrospray‐carbonization strategy, SiO*_x_* nanoparticles (NPs) are encapsulated in the nitrogen‐doped carbon (N–C) frameworks decorating atomically dispersed iron sites. Systematic characterizations including high‐angle annular dark‐field scanning transmission electron microscopy (HAADF‐STEM) and X‐ray absorption fine structure (XAFS) verify the existence of Fe single atoms and typical coordination environment. Benefiting from its structural and compositional merits, the SiO*_x_*/Fe–N–C anode delivers significantly improved discharge capacity of 799.1 mAh g^−1^, rate capability, and exceptional durability, compared with pure SiO_2_ and SiO*_x_*/N–C, which has been revealed by the density functional theory (DFT) calculations. Additionally, the electrochemical tests and in situ X‐ray diffraction (XRD) analysis reveal the oxidation of Li*_x_* Si phase and the storage mechanism. The synthetic strategy is universal for the design and synthesis of metal single atoms/clusters dispersed N–C frameworks encapsulated SiO*_x_* NPs. Meanwhile, this work provides impressive insights into developing various LIB anode materials suffering from inferior conductivity and huge volume fluctuations.

## Graphical abstract

A universal and facile electrospray‐carbonization strategy is adopted for synthesizing silicon oxide/iron‐nitrogen co‐doped carbon (SiO*_x_*/Fe–N–C) materials, which utilizes sustainable rice husks as silicon sources. The Fe–N–C frameworks decorating atomic dispersed Fe sites are employed as the disperse medium of SiO*_x_* nanoparticles, facilitating the ion diffusion and charge transfer, accommodating the volume change, and electrochemical reversibility for lithium storage.

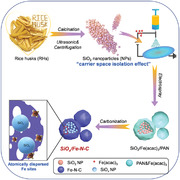

## 1 Introduction

Recently, nitrogen–doped carbon (N–C) frameworks supported transition metal (M = Fe,^[^ ^1^, ^2^ ^]^ Co,^[^ ^3^ ^]^ and Ni^[^ ^4^ ^]^) single atom or cluster catalysts possess good electrochemical properties, because of satisfactory conductivity and electrochemical activity. In addition, metal–nitrogen–carbon (M–N–C) can promote the adsorption/desorption of active sites, manifesting its applications in oxygen reduction reaction or even metal (Zn, Al, and Li)‐air batteries.^[^ ^5^ ^]^ M–N–C can also be utilized as sulfur hosts of Li‐S batteries with desirable capacity and superior cyclability.^[^ ^6^ ^]^ The presence of M–N–C with catalytic attribute can significantly relieve the “shuttle effect” of polysulfide produced during cycling, and further accelerate the formation and decomposition of Li_2_S/Na_2_S for Li/Na‐S batteries.^[^ ^7^, ^8^ ^]^ Li‐ion batteries (LIBs) are employed as attractive power sources for multifarious energy storage systems.^[^ ^9^ ^]^ It is of great significance whether the M–N–C‐based anodes with the catalytic effect can enhance the reversibility of electrochemical reactions during lithium storage and reveal the corresponding interaction between Li^+^ and M–N–C.^[^ ^10^ ^]^

The alternative silicon suboxide (SiO*_x_*) (0 < *x* ≤ 2) anodes have also been widely investigated for LIB applications because of rich abundance of Si, high capacity, easy synthesis, and low cost.^[^ ^11^, ^12^ ^]^ Despite above superiorities, the drawbacks such as obvious volume fluctuations and inferior conductivity of SiO*_x_* still lead to undesirable cycle stability and rate performance.^[^ ^13^ ^]^ To solve afore‐mentioned problems, a general strategy is to construct SiO*_x_* composites with metal (Cu,^[^ ^14^ ^]^ Ni,^[^ ^15^ ^]^ Co,^[^ ^16^ ^]^ Ag,^[^ ^17^ ^]^ etc.) or/and carbon materials, directly bringing about increased electronic conductivity. Besides, electrochemically inert metals can also serve as buffer space for accommodating volume expansion.^[^ ^18^ ^]^ Another strategy for relieving volume effect is to construct core–shell, yolk–shell, hollow structures as well as carbon frameworks.^[^ ^19^ ^]^ In addition, achieving a balance between decreasing the size of SiO*_x_* as electroactive component and reducing the agglomeration degree of nanostructures can improve its electrochemical activity, reduce the volume strain, and decrease the inter‐particle resistance induced by ineffective contact.^[^ ^20^ ^]^ Thus, it is of great significance to design nano‐SiO*_x_* composites with desirable structures.

In terms of Si sources, biomass resources (bamboo leaves,^[^ ^15^ ^]^ reed leaves,^[^ ^21^ ^]^ rice husks (RHs),^[^ ^22^ ^]^ etc.) can provide sustainable and abundant SiO_2_ precursors for further synthesis of SiO*_x_*‐based materials. Herein, we utilized rice husks as SiO_2_ precursors and adopted a combined strategy of electrospray‐carbonization processes to prepare SiO*_x_*/Fe–N–C materials. SiO*_x_* NPs with good dispersion were encapsulated in Fe single atoms decorated N–C frameworks. The aberration‐corrected high‐angle annular dark‐field scanning transmission electron microscopy (HAADF‐STEM) and X‐ray absorption fine structure (XAFS) tests verified the presence of Fe single atoms and Fe–N coordination mode. When SiO*_x_*/Fe–N–C was used as LIB anodes, it exhibited high discharge capacities of 799.1 mAh g^−1^ at the 100th cycle and 173.7 mAh g^−1^ at the 5000th cycle even at 5 A g^−1^. By combining density functional theory (DFT) calculations, in situ X‐ray diffraction (XRD) technique and ex situ morphology and structural characterizations, we concluded the reasons for significantly improved performance of the SiO*_x_*/Fe–N–C anode and revealed the oxidation of Li*_x_* Si phase as well as the storage mechanism. Its unique structural and compositional advantages rendered efficient active sites for lithium storage, promoted its electrochemical reversibility with the catalytic attribute of Fe single atoms, and buffered its volume fluctuations of SiO*_x_* NPs. The rational syntheses of SiO*_x_*/M–N–C (M = Fe, Co, Ni) demonstrated that such strategy could be extended for fabricating other SiO*_x_*‐based composites or M–N–C materials. In view of the effect of M–N–C on lithium storage, these materials are expected for diverse applications.

## 2 Results and Discussion

**Figure 1:**
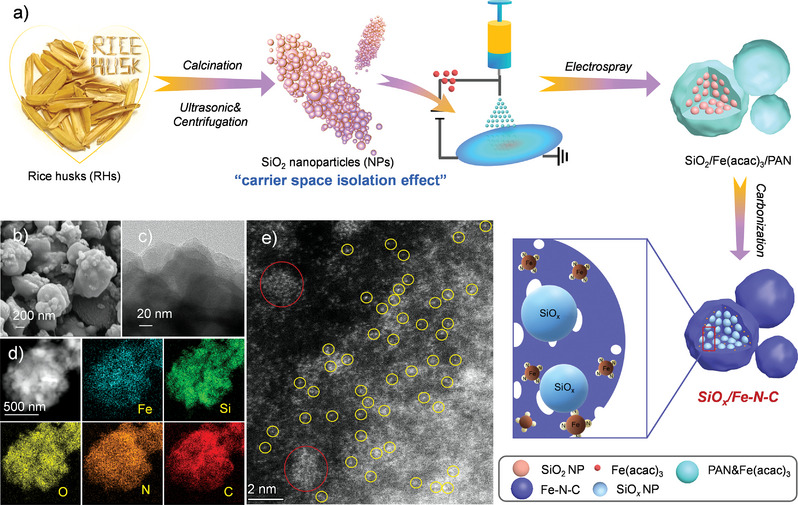
a) Schematic illustration of the fabrication of SiO*_x_*/Fe–N–C; b,c) Scanning electron microscopy (SEM) and transmission electron microscope (TEM) images of SiO*_x_*/Fe–N–C; d) Energy dispersive spectrometer (EDS)‐mapping result and e) Aberration‐corrected HAADF‐STEM result of the as‐synthesized SiO*_x_*/Fe–N–C product.

The whole synthetic procedure for preparing the SiO*_x_*/Fe–N–C product is presented in **Figure** **1**a. RHs was initially washed and acid treated for subsequent calcination to obtain SiO_2_ powder, of which the powder was mainly composed of biomass skeleton and SiO_2_ NPs (Figure S1a,b, Supporting Information). Furthermore, SiO_2_ NPs with average particle sizes of 20–80 nm can be easily separated with the assistance of ultrasonic and centrifugal processes (Figure S1c,d, Supporting Information), showing a lower aggregation degree compared with other nanosized SiO_2_.^[^ ^22^ ^]^ This is crucial for preparing carbon coated SiO*_x_* composites. Afterward, the precursor solution containing polyacrylonitrile (PAN), SiO_2_ NPs, and Fe(acac)_3_ was used to produce SiO*_x_*/Fe–N–C microspheres through the electrospray‐carbonization strategy. For comparison, the precursor solution without adding Fe(acac)_3_ was denoted as SiO*_x_*/N–C.

The as‐synthesized SiO*_x_*/Fe–N–C composites have an irregular spherical morphology with average sizes of ≈0.5–1 µm (Figure 1b). Similarly, the SiO*_x_*/N–C sample exhibited a similar micro‐spherical morphology and size with SiO*_x_*/Fe–N–C (Figure S2, Supporting Information). Furthermore, SiO*_x_* NPs were enwrapped by the micro‐spherical framework, and no obvious metallic Fe NPs were detected from its edge of spherical framework (Figure 1c). Moreover, the high‐resolution transmission electron microscopy (HRTEM) and selected area electron diffraction (SAED) images demonstrated that no obvious crystal lattice and diffraction fringes could be observed (Figure S3, Supporting Information), indicating that the components of SiO*_x_* and carbon were both amorphous.^[^ ^23^ ^]^ Theoretically, the product synthesized by the electrospray‐carbonization strategy should contain Fe, Si, O, C, and N elements, as revealed by the energy dispersive spectrometer (EDS)‐mapping images (Figure 1d). The Fe element was homogeneously distributed in SiO*_x_*/Fe–N–C. Therefore, the existing form of Fe species is of great significance to be investigated by the HAADF‐STEM technique. For SiO*_x_*/Fe–N–C, Fe single atoms (yellow circles) and a small number of atomic clusters (red circles) could be detected rather than Fe NPs (Figure 1e). Fe single atoms and clusters mainly appeared near the edge of the microspheres and around SiO*_x_* NPs, respectively. The mass loadings of Fe (≈6 wt%), N–C (≈43 wt%), and SiO*_x_* (≈51 wt%) in SiO*_x_*/Fe–N–C were determined by a combination of thermogravimetric analysis (TGA) (Figure S4, Supporting Information), inductively coupled plasma optical emission spectroscopy (ICP‐OES), and X‐ray photoelectron spectroscopy (XPS) results (Table S1, Supporting Information). The synthetic strategy is of great significance for obtaining relatively high mass loading of metal single atoms. The synthetic basis for introducing Fe single atoms can be ascribed to the carrier space isolation effect of SiO_2_ or SiO*_x_* NPs.^[^ ^24^ ^]^ Apart from reducing the aggregation of Fe salts, the presence of N–C promoted the formation of Fe–N active sites at the molecular level after carbonization,^[^ ^25^ ^]^ which was subsequently proved by the XAFS characterization.

Meanwhile, since the metal salts were almost dissolved in the precursor solution, the formation of Fe clusters in N–C was inevitable due to the inside distribution of SiO*_x_* NPs.^[^ ^26^ ^]^ Theoretically, the catalytic activity of atomic and clusters is much higher than that of Fe NPs. Meanwhile, we have tried to reduce the formation of Fe clusters by increasing the concentration of SiO*_x_* NPs. However, it is difficult to obtain the electrospray products with good carbon coating since the concentration of PAN is very low, which could lead to serious agglomeration of SiO*_x_* NPs and poor electrochemical activity for lithium storage. Herein, we have proved that Fe element in SiO*_x_*/Fe–N–C mainly existed as Fe single atoms rather than atomic clusters around SiO*_x_* as described above. Therefore, it is focused on investigating whether Fe single atoms have an elevating effect on the reversibility of SiO*_x_* during cycling, and the DFT calculations involving the influence of Fe single atoms.

To analyze the influence of carbonization temperature on the detailed Fe species, the basic morphology characterization of the C1 and C2 samples were conducted. The overall spherical morphology of C1 and C2 was similar with that of SiO*_x_*/Fe–N–C. No obvious Fe NPs displayed in the TEM, HRTEM, and SAED images could be found (Figure S5a–e, Supporting Information), and Fe element was evenly distributed in the EDS‐mapping results (Figure S5f, Supporting Information). This result was similar with that of SiO*_x_*/Fe–N–C, which implied that the Fe element was successfully doped in the N–C framework. Instead, when the carbonization temperature was determined to be 750 °C, Fe NPs were strongly distinguished by the TEM, HRTEM, and SAED images of the C2 sample (Figure S6a–e, Supporting Information). The size of the Fe NPs was obviously over 20 nm, and the distribution of Fe element could confirm that the Fe species of C2 were metallic NPs (Figure S6f, Supporting Information), which was also revealed by the XRD curve (Figure S6g, Supporting Information). In short, the Fe species changed from single atoms/clusters to NPs when elevating the carbonization temperature, showing an apparent agglomeration tendency of atoms. When choosing suitable carbonization temperature, the N–C species decomposed from PAN could subsequently bind with Fe atoms to generate Fe–N*_x_* moieties, inhibiting Fe atoms from serious aggregation.^[^ ^27^, ^28^ ^]^

**Figure 2:**
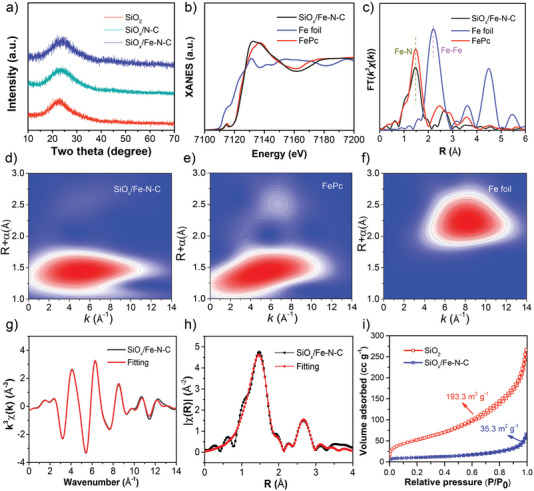
a) XRD curves of pristine SiO_2_, SiO*_x_*/N–C, and SiO*_x_*/Fe–N–C. The SiO*_x_*/Fe–N–C, reference Fe foil and FePc samples. b) XANES results; c) FT‐EXAFS results; d–f) WT‐EXAFS analysis; g,h) The corresponding EXAFS K‐space and R‐space fitting curves of SiO*_x_*/Fe–N–C; i) N_2_ absorption–desorption isotherms of the pristine SiO_2_ and SiO*_x_*/Fe–N–C samples.

XRD patterns of the three samples displayed a broad peak located between 20° and 30° (**Figure** **2**a), corresponding to amorphous SiO_2_, SiO*_x_*, and N–C.^[^ ^29^, ^30^ ^]^ No obvious signals of Fe and FeO*_x_* were found, excluding the existence of good crystalline component of Fe‐containing species. Besides, the Raman spectra also exhibited two typical D‐band (1353 cm^−1^) and G‐band (1591 cm^−1^) for carbon (Figure S7, Supporting Information).^[^ ^31^ ^]^ Furthermore, we employed XAFS technique for its detailed analysis of chemical state and corresponding coordination environment of the SiO*_x_*/Fe–N–C product. Figure 2b displays the X‐ray absorption near edge structure (XANES) spectra of SiO*_x_*/Fe–N–C and their reference samples of Fe foil and FePc. The energy absorption threshold of SiO*_x_*/Fe–N–C was closer to FePc at the Fe K‐edge, indicating that the Fe^*δ*+^ with positive charge was stabilized by N atoms.^[^ ^32^ ^]^ In addition, the pre‐edge peak of SiO*_x_*/Fe–N–C located at about 7114.2 eV was consistent with the representative Fe–N_4_ structure.^[^ ^33^ ^]^ Figure 2c provides the Fourier transform extended‐XAFS (FT‐EXAFS) results. For SiO*_x_*/Fe–N–C, the existence of Fe—N bond (1.48 Å) demonstrated that Fe atoms were primarily coordinated with N atoms rather than other Fe atoms (Fe—Fe bond at about 2.20 Å), which was identical with FePc.^[^ ^34^ ^]^ Wavelet transform (WT) can also be employed for the analysis of the Fe K‐edge EXAFS oscillations. The WT‐EXAFS result of SiO*_x_*/Fe–N–C displayed an obvious intensity maximum of ≈5.0 Å^–1^, which was close to that of FePc and completely distinct from that of reference Fe foil (≈8.0 Å^–1^) (Figure 2d–f).^[^ ^35^ ^]^ The detailed chemical configuration of Fe was further revealed by the corresponding FT‐EXAFS fittings in R and k spaces. As exhibited in Figure 2g,h and Figure S8 (Supporting Information), the average coordination number (4.8) and bond length (1.92 Å) for Fe–N in SiO*_x_*/Fe–N–C were found (Table S2, Supporting Information).^[^ ^36^ ^]^ Based on the afore‐mentioned HAADF‐STEM and XAFS results, the Fe single atoms as primary Fe species in SiO*_x_*/Fe–N–C and the existence of Fe—N bonds were confirmed.

Additionally, the XPS measurement of SiO*_x_*/Fe–N–C was also employed for the analysis of its chemical compositions. All elements were involved in SiO*_x_*/Fe–N–C (Figure S9a, Supporting Information). The N 1s spectrum showed that it mainly involved pyridinic (398.6 eV), pyrrolic (400.2 eV), graphitic (401.2 eV) nitrogen, and Fe–N (399.6 eV) species, providing abundant sites for fixing Fe atoms (Figure S9b, Supporting Information).^[^ ^37^ ^]^ The Si 2p spectrum could be fitted into the characteristic peaks of Si^2+^, Si^3+^, and Si^4+^ (Figure S9c, Supporting Information). Compared to pure SiO_2_ (Figure S10, Supporting Information), three different valences of Si could be observed, which was caused by the reduction of N–C on Si^4+^ in SiO_2_ at high temperature. Besides, the corresponding O 1s, C1s, and Fe 2p spectra results were provided in Figure S9d–f (Supporting Information). For comparison, the XPS results of C1 and C2 (Figures S11 and S12, Supporting Information) were consistent with the afore‐mentioned TEM and HRTEM results. The SiO*_x_*/Fe–N–C sample showed a much smaller specific surface area (35.8 m^2^ g^−1^), compared with that of the SiO_2_ precursor (193.3 m^2^ g^−1^) (Figure 2i). This was attributed to that SiO*_x_* NPs were compactly wrapped by Fe–N–C, of which the frameworks could effectively reduce the side reactions resulting from its direct exposure of SiO*_x_* NPs and thus promoted capacity and cyclability.^[^ ^38^ ^]^ Meanwhile, SiO*_x_*/Fe–N–C had a smaller pore volume (0.1 cm^3^ g^−1^) and a broad pore size distribution (2–10 nm) (Figure S13, Supporting Information), which ensured reversible intercalation and deintercalation of Li^+^ in SiO*_x_*/Fe–N–C.^[^ ^23^ ^]^

**Figure 3:**
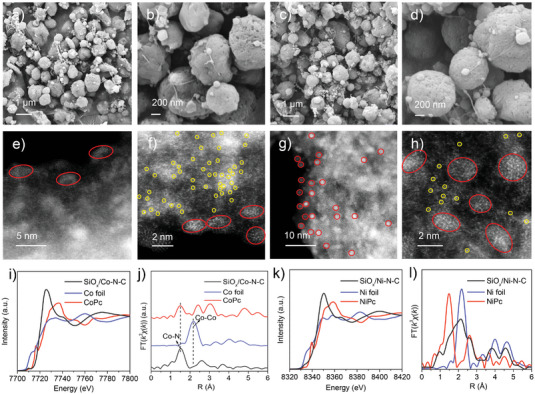
Universal synthesis of SiO*_x_*/Co–N–C and SiO*_x_*/Ni–N–C. a,b) SEM images of SiO*_x_*/Co–N–C; c,d) SEM images of SiO*_x_*/Ni–N–C; e,f) HAADF‐STEM images of SiO*_x_*/Co–N–C; g,h) HAADF‐STEM images of SiO*_x_*/Ni–N–C; i,j) XANES and FT‐EXAFS analysis of SiO*_x_*/Co–N–C, reference Co foil and CoPc; k,l) XANES and FT‐EXAFS results of the SiO*_x_*/Ni–N–C sample, reference Ni foil and NiPc.

Based on above morphology and structural characterizations of SiO*_x_*/Fe–N–C, the synthetic strategy could also be extended for obtaining SiO*_x_*/Co–N–C and SiO*_x_*/Ni–N–C. The products synthesized by electrospray‐carbonization strategy showed similar spherical morphology with diameters of 0.5–2 µm (**Figure** **3**a–d). The HAADF‐STEM image of SiO*_x_*/Co–N–C displayed that the Co species mainly exist as Co single atoms (yellow circle) and small amounts of Co clusters (red circle) appeared near the edge of the microspheres (Figure 3e,f). For SiO*_x_*/Co–N–C, the apparent pre‐edge peak at ≈7109.9 eV could be detected by the XANES result, suggesting a square‐planar Co–N_4_ structure (Figure 3i). Co atoms were coordinated with N atoms rather than other Co atoms, which were identical with CoPc and consistent with the dominant presence of Co singles atoms (Figure 3j). Unlike SiO*_x_*/Co–N–C, large amounts of Ni clusters (red circle) of ≈2 nm in size, and small amounts of Ni single atoms (yellow circle) were observed (Figure 3g,h). The corresponding XANES and FT‐EXAFS analysis confirmed its composition and obvious existence of Ni clusters (Figure 3k,l). Overall, the electrospray‐carbonization method was reasonable and universal for the designing SiO*_x_*/M–N–C or M–N–C materials, which could be further applied in various rechargeable batteries or electrocatalysts.

**Figure 4:**
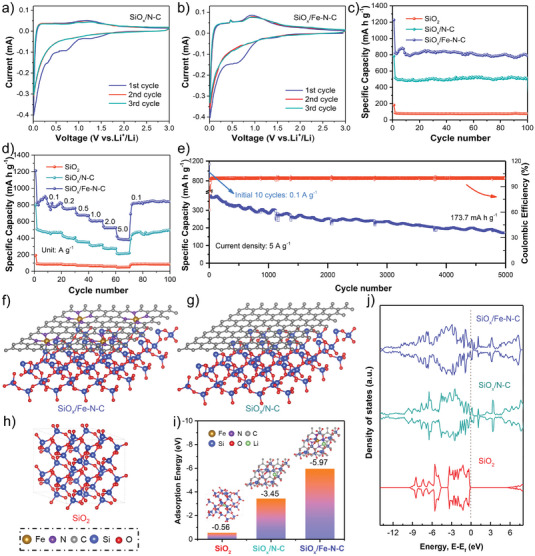
a,b) CV curves of SiO*_x_*/N–C and SiO*_x_*/Fe–N–C, respectively; c) Cycling stability of the SiO_2_, SiO*_x_*/N–C and SiO*_x_*/Fe–N–C samples at 100 mA g^−1^; d) Rate performance of SiO_2_, SiO*_x_*/N–C, and SiO*_x_*/Fe–N–C; e) Long‐term cyclability of SiO*_x_*/Fe–N–C at 5 A g^−1^. DFT calculations of the SiO*_x_*/Fe–N–C, SiO*_x_*/N–C, and *α*‐SiO_2_ samples. f–h) The calculated geometry configurations; i) The calculated Li adsorption energy; j) The DOS results.

To measure the lithium storage performance and of pure SiO_2_, SiO*_x_*/N–C, and SiO*_x_*/Fe–N–C materials as LIB anodes and the effect of different compositions, the representative 2032 coin‐type cells based on lithium foil as the counter electrode were assembled. **Figure** **4**a,b and Figure S14a (Supporting Information) display the above three cyclic voltammogram (CV) curves, respectively. Taking SiO*_x_*/Fe–N–C as an example, the reduction peaks at 1.43 and 0.57 V were detected during the 1st cathodic process and disappeared in the later cycles, which were assigned by the decomposition of electrolyte and the generation of the solid electrolyte interface (SEI) film.^[^ ^39^ ^]^ A remaining steep peak of ≈0.01 V resulted from its alloying reaction of Si and Li^+^ for the generation of Li*_x_* Si.^[^ ^40^ ^]^ Notably, in the 1st anodic curve, the existence of 0.47 V represented the dissociation process of Li*_x_* Si.^[^ ^41^, ^42^ ^]^ A broad anodic peak (≈0.92 V) corresponded to the Li^+^ extraction of Li‐containing components (Li_2_Si_2_O_5_, LiC*_x_*).^[^ ^43^, ^44^ ^]^ The remaining 2nd and 3rd cycles with no distinct changes demonstrated its superb reversibility. In general, compared with the anodic scans of SiO*_x_*/M–N–C (M = Fe, Co, Ni), the dissociation peak of Li*_x_* Si could not be directly detected in that of SiO*_x_*/N–C (Figure S14b,c, Supporting Information). Moreover, the SiO*_x_*/N–C sample displayed a wider range, higher potential and lower current intensity of the dissociation peak (≈1.2 V). Above results could be related to the introduction of metal single atoms with catalytic attribute into the N–C frameworks, promoting the dissociation of amorphous Li*_x_* Si alloy and the utilization of Si for boosted electrochemical reversibility.^[^ ^10^, ^45^ ^]^

Figure S15 (Supporting Information) shows the classic discharge/charge curves of the SiO_2_, SiO*_x_*/N–C, and SiO*_x_*/Fe–N–C anodes at 100 mA g^−1^. The SiO*_x_*/Fe–N–C anode delivered 1st discharge/charge capacities of 1226.9/758.3 mAh g^−1^ along with a 61.8% coulombic efficiency. Different discharge capacities of 821.3, 820.4, and 821.9 mAh g^−1^ were achieved for the optimized SiO*_x_*/Fe–N–C anode after 2, 20, and 50 cycles, respectively. SiO*_x_*/Fe–N–C exhibited much superior capacities than SiO_2_ and SiO*_x_*/N–C. Meanwhile, the discharge capacities of 78.6, 515.4, and 799.1 mAh g^−1^ were achieved at the 100th cycle for pure SiO_2_, SiO*_x_*/N–C, and SiO*_x_*/Fe–N–C, respectively (Figure 4c). Only a decrease of capacity (22.2 mAh g^−1^) and a 2.7% capacity decay from the 2nd to the 100th cycle was retained for SiO*_x_*/Fe–N–C, illustrating a critical role of the SEI film during cycling on maintaining the discharge capacities.^[^ ^13^ ^]^ Figure 4d and Figure S16 (Supporting Information) show the rate performance. The SiO*_x_*/Fe–N–C product delivered reversible discharge capacities of 801.4, 750.7, 677.8, 613.2, 532.0, and 386.2 mAh g^−1^ at 0.1, 0.2, 0.5, 1.0, 2.0, and 5.0 A g^−1^, respectively, and achieved 826.7 mAh g^−1^ when its current recovered to 0.1 A g^−1^. Compared with the pure SiO_2_ and SiO*_x_*/N–C samples, the rate capability of SiO*_x_*/Fe–N–C was significantly enhanced, demonstrating that the conductivity was promoted and the result was later proved by the density of states (DOS) calculation. It was noted that the superior lithium storage performance of the as‐obtained SiO*_x_*/Fe–N–C composite was highly comparable among those of various SiO*_x_*‐based anodes (Figure S17, Table S3, Supporting Information).

The electrochemical impedance spectroscopy (EIS) measurements were also provided for investigating the effect of Fe–N–C on improved rate capability before cycling. In the EIS curves of the three samples, the typical high‐frequency semicircles and the low‐frequency inclined line can reveal the charge‐transfer resistance (*R*_ct_) and the diffusion process of Li^+^, respectively (Figure S18, Supporting Information). SiO*_x_*/Fe–N–C had a much smaller *R*_ct_ value (72.1 Ω) than SiO*_x_*/N–C (118.2 Ω) and fresh SiO_2_ (165.8 Ω), revealing that introducing Fe–N–C was more efficient for enhancing the conductivity of SiO*_x_*.^[^ ^46^ ^]^ Besides, the optimized SiO*_x_*/Fe–N–C sample displayed superior long‐term cycle performance with a discharge capacity of 173.7 mAh g^−1^ after 5000 cycles at 5 A g^−1^, as indicated in Figure 4e.

At the same time, to assess the lithium adsorption abilities of the optimized SiO*_x_*/Fe–N–C product, the first‐principles DFT calculations of adsorption energy for pristine SiO_2_, SiO*_x_*/N–C, and SiO*_x_*/Fe–N–C was performed. Notably, the effect of Fe single atoms on lithium storage performance was mainly considered. The valence of Si element in SiO*_x_*/N–C or the optimized SiO*_x_*/Fe–N–C sample was close to the valence of Si in SiO_2_. Thus, according to the ever‐reported references, the three models were constructed (Figure 4f–h). As a result, the lithium adsorption energies were determined to be −0.56, −3.45, and −5.97 eV for the pure SiO_2_, SiO*_x_*/N–C, and SiO*_x_*/Fe–N–C products, respectively (Figure 4i). SiO*_x_*/Fe–N–C possessed the strongest adsorption ability, which was consistent with the highest discharge capacity.^[^ ^47^, ^48^ ^]^ In addition, the DOS calculation was employed to investigate the electrical conductivity (Figure 4j). The introduction of Fe–N–C significantly improved the conductivity of SiO*_x_* resulting from the largest density difference at Fermi level.^[^ ^1^ ^]^ Therefore, Fe–N–C can exhibit the greatest potential in achieving the best capacity and rate capability for lithium storage.

To study the charge storage processes of SiO*_x_*/Fe–N–C, the CV tests were carried out at diverse rates of 0.2–1.0 mV s^−1^ (Figure S19a, Supporting Information). Generally, their logarithmic values of the scan rates (*v*) and the peak currents (*i*) conformed to the equations (*i* = a*v* ^b^; log*i* = *b*log*v* + log*a*). Among a and b acting as the regulable parameters, the *b*‐values of 0.5 and 1.0 can stand for an ideal diffusion/pseudocapacitive‐controlled processes, respectively.^[^ ^49^ ^]^ The *b*‐values of 0.68 and 0.81 were obtained for SiO*_x_*/Fe–N–C (Figure S19b, Supporting Information), revealing that its charge storage process was determined by diffusion‐ and pseudocapacitive‐controlled behaviors.^[^ ^50^ ^]^ In addition, the pseudocapacitive contributions at varied rates for SiO*_x_*/Fe–N–C were displayed and their values of 61.3%, 65.9%, 72.8%, 75.3%, and 77.2% were achieved, respectively (Figure S20a–f, Table S4, Supporting Information). The SiO*_x_*/Fe–N–C anode showed much higher values than those of non‐metal doped SiO*_x_*/N–C (Figures S21 and S22, Supporting Information), indicating Fe‐doping promoted its electrochemical reaction and storage reversibility.

**Figure 5:**
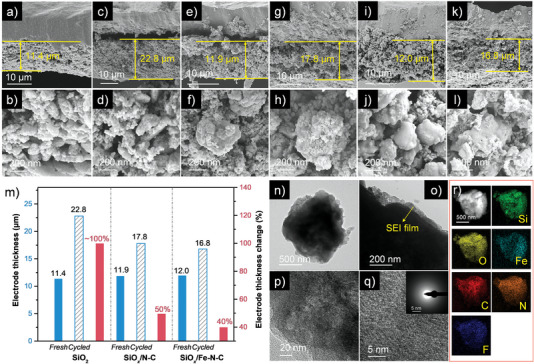
SEM images. a,b) SiO_2_ before cycling; c,d) SiO_2_ after cycling; e,f) SiO*_x_*/N–C before cycling; g,h) SiO*_x_*/N–C after cycling; i,j) SiO*_x_*/Fe–N–C before cycling; k,l) SiO*_x_*/Fe–N–C after cycling; m) Bar chart of the electrode thickness and corresponding change rate for the fresh and cycled SiO_2_, SiO*_x_*/N–C, and SiO*_x_*/Fe–N–C anodes; n–p) TEM images of the cycled SiO*_x_*/Fe–N–C sample; q) HRTEM and inserted SAED images of the SiO*_x_*/Fe–N–C anode after cycling; r) EDS‐mapping patterns of SiO*_x_*/Fe–N–C after cycling.

Apart from basal electrochemical tests, the structural stability of SiO*_x_*/Fe–N–C was further analyzed by the SEM characterizations. The results of comparable pure SiO_2_ and SiO*_x_*/N–C were also given. The cross‐sectional thickness of the pure SiO_2_ anode increased from 11.4 to 22.8 µm after cycling, and the agglomerated particle structure were obviously damaged (**Figure** **5**a–d).^[^ ^51^ ^]^ By contrast, the thickness of SiO*_x_*/Fe–N–C increased from 12.0 to 16.8 µm, and the micro‐spherical structure could be well observed after 100 cycles (Figure 5e–h), of which the thickness change was smaller than that of SiO*_x_*/N–C (Figure 5i–m). Figure 5n–q demonstrates the presence of stable SEI film and its good structural integrity of the cycled SiO*_x_*/Fe–N–C anode.^[^ ^52^ ^]^ Moreover, the amorphous properties of SiO*_x_*/Fe–N–C after cycling were not changed and no obvious Fe NPs could be detected. The EDS‐mapping result also showed the existence of F element, resulting from LiF as a component of the indispensable SEI film (Figure 5r). Above results illustrated that Fe–N–C efficiently relieved the volume fluctuations upon cycling and maintained its structural stability.

**Figure 6:**
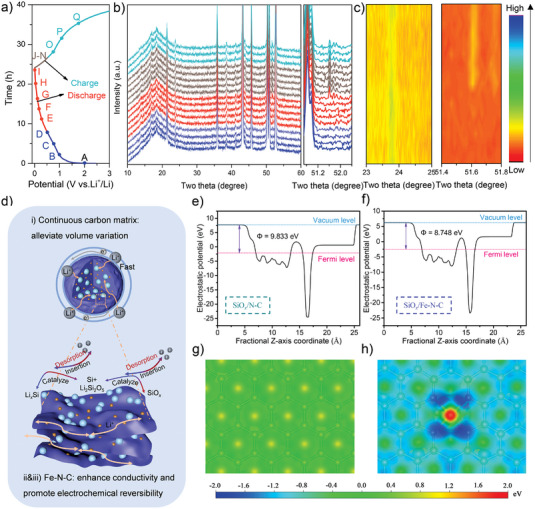
a,b) In situ XRD curves under different voltages and the corresponding charge/discharge curves of SiO*_x_*/Fe–N–C; c) The corresponding contour plots of SiO*_x_*/Fe–N–C; d) Schematic illustration of the reversible conversion‐alloy reaction for boosted lithium storage performance of SiO_x_/Fe–N–C during the discharging/charging processes; e,f) The work functions for SiO*_x_*/N–C and SiO*_x_*/Fe–N–C, respectively; The calculated electron‐density isosurface for g) SiO*_x_*/N–C and h) SiO*_x_*/Fe–N–C, respectively. The electron‐density isosurface was plotted at 0.01 e bohr^−3^. The color bar represented the electrostatic potential scale.

More importantly, to study its lithium storage mechanism of the as‐synthesized SiO*_x_*/Fe–N–C anode, the in situ XRD measurements were performed. The XRD curves under different voltages and the corresponding charge/discharge curves (0.01–3.0 V) are displayed in **Figure** **6**a,b, respectively. As a result, the diffraction peaks involved the typical signals of Be foil (45.9°, 50.9°, 52.8°), BeO (38.6°, 41.3°, 43.8°), Li foil (35.9°, 51.9°), and Cu foil (43.3°), which were not participated in the electrochemical reaction upon cycling.^[^ ^53^ ^]^ The corresponding contour plots are displayed in Figure 6c. It is notable that the peak at 23.8° in the contour plot was related with the formation of Li*_x_* Si and the peak at 51.7° could be assigned to the formation of Si, mainly resulting from the dissociation of amorphous Li*_x_* Si, which were consistent with the CV curves.^[^ ^54^, ^55^, ^56^ ^]^ The signal of Si became weak but still existed when the voltage was charged to 3.0 V. This is because the generation of Si during the first discharge process cannot completely convert into SiO*_x_* (SiO*_x_* + 2*x*Li^+^ + 2*x*e^−^ → *x*Li_2_O + Si; SiO*_x_* + *x*Li^+^ + *x*e^−^ → 0.25*x*Li_4_SiO_4_ + (1–0.25*x*)Si; SiO*_x_* + 0.4*x*Li^+^ + 0.4*x*e^−^ ↔ 0.2*x*Li_2_Si_2_O_5_ + (1–0.4*x*)Si; Si + *x*Li^+^ + *x*e^−^ ↔ Li*_x_* Si (*x* ≤ 4.4).^[^ ^57^ ^]^ Normally, partial Li_2_Si_2_O_5_ can transform into SiO*_x_*. Thus, the reversible formation and dissociation of Li*_x_* Si could improve the utilization of Si and promote the electrochemical reversibility.^[^ ^45^ ^]^ Furthermore, the most probable conversion‐alloy reaction mechanism of SiO*_x_* in SiO*_x_*/Fe–N–C composite was confirmed, as schematically indicated in Figure 6d.

In addition, the work function is close related to the electrochemical properties of its material surface and can reflect the relative difficulty level of the electron escaping from the surface. The calculations of the work functions for the SiO*_x_*/N–C and as‐obtained SiO*_x_*/Fe–N–C samples were performed and their values of 9.833 and 8.748 eV were determined (Figure 6e,f). The lower value of the work function for SiO*_x_*/Fe–N–C was beneficial for promoting its electron transfer and catalyze the electrochemical reactions such as the dissociation/formation of amorphous Li*_x_* Si.^[^ ^58^, ^59^ ^]^ Moreover, the electron‐density isosurface of SiO*_x_*/Fe–N–C also verified higher ability to transfer electrons compared with SiO*_x_*/N–C (Figure 6g,h).^[^ ^60^, ^61^ ^]^

The enhanced performances were attributed to the reasons: 1) Fe–N–C frameworks efficiently prevented the accumulation of SiO*_x_* NPs and improved the electrochemical activity of SiO*_x_*; 2) Fe–N–C could both increase the conductivity and effectively alleviate the volume fluctuations caused by nano‐SiO*_x_* upon cycling; 3) Fe single atoms with catalytic attribute promoted the dissociation of Li*_x_* Si phase and enhanced its electrochemical reversibility.

## 3 Conclusion

In summary, a simple electrospray‐carbonization strategy for obtaining SiO*_x_*/Fe–N–C materials was developed. Atomically dispersed Fe sites embedded within spherical N–C frameworks was used as a good dispersion medium of SiO*_x_* NPs. It was noted that Fe single atoms as the main existence form in SiO*_x_*/Fe–N–C were indeed proved by the HAADF‐STEM and XAFS results. According to the experimental and DFT results, the presence of Fe–N–C frameworks not only boosted the conductivity, accommodated the volume changes of SiO*_x_*, but also promoted the reversibility of Li*_x_* Si, resulting in significantly improved electrochemical performance of SiO*_x_*/Fe–N–C. Additionally, its lithium storage mechanism was also proved by the in situ XRD results. Finally, this work presented an efficient strategy to rationally fabricate metal single atoms/clusters decorating carbon/nano‐SiO*_x_* composite anodes for high‐performance LIBs, which was also expected for other energy storage and conversion applications.

## Conflict of Interest

The authors declare no conflict of interest.

## Supporting information

Supporting Information

## Data Availability

The data that support the findings of this study are available from the corresponding author upon reasonable request.
