# Supplementary material for: Embedding Atomically Dispersed Iron Sites in Nitrogen‐Doped Carbon Frameworks‐Wrapped Silicon Suboxide for Superior Lithium Storage
Source: Adv Sci (Weinh). 2022 Dec 5;10(4):2206084. doi: 10.1002/advs.202206084 (PMC9896072; doi:10.1002/advs.202206084)
Supplement: Supplementary file 1 — Supporting Information [file ADVS-10-2206084-s001.pdf]

ADVANCED SCIENCE
Open Access

Supporting Information

for *Adv. Sci.*, DOI 10.1002/advs.202206084

Embedding Atomically Dispersed Iron Sites in Nitrogen-Doped Carbon Frameworks-Wrapped Silicon Suboxide for Superior Lithium Storage

*Xiaotian Guo, Hengyue Xu, Wenting Li, Yangyi Liu, Yuxin Shi, Qing Li and Huan Pang**

Supporting Information

Embedding Atomically Dispersed Iron Sites in Nitrogen-doped Carbon Frameworks-Wrapped Silicon Suboxide for Superior Lithium Storage

Xiaotian Guo†*, Hengyue Xu*†*, Wenting Li, Yangyi Liu, Yuxin Shi, Qing Li, and Huan Pang**

† These authors contribute equally.

Dr. X. Guo, Dr. W. Li, Dr. Y. Liu, Dr. Y. Shi, Prof. H. Pang
School of Chemistry and Chemical Engineering, Yangzhou University, Yangzhou, Jiangsu, 225009, P. R. China
E-mail: huanpangchem@hotmail.com; panghuan@yzu.edu.cn

Mr. H. Xu
Institute of Biopharmaceutical and Health Engineering, Tsinghua Shenzhen International Graduate School, Tsinghua University, Shenzhen 518055, P. R. China

Prof. Q. Li
Guangling College, Yangzhou University, Yangzhou, 225009, Jiangsu, P. R. China.

Experimental Section

1.1 Materials and reagents.

All chemicals were purchased from Shanghai Sinopharm Chemical Reagent, co. ltd., and used without further treatment or purification. All aqueous solutions were prepared with high-purity de-ionized water. (DI-water, resistance 18 MΩ cm^{-1}).

1.2 Synthesis

1.2.1 Synthesis of SiO_2

For rice husks (RHs)-derived SiO_2 nanoparticles (NPs), the RHs collected from the rice mill in Yangzhou City were washed several times with distilled water and immersed in 3 mol L^{-1} hydrochloric acid (HCl) solution. The acid treated RHs collected by filtration were repeatedly washed with distilled water and after drying in an oven at 80 °C overnight, the powder was annealed at 700 °C for 6 h in air at a heating rate of 3 °C min^{-1} to remove the organic components (cellulose, lignin) and thus obtain white-beige SiO_2 powder. The SiO_2 power was grinded and separated into 300 mesh powder, immersed in a certain amount of water and ultrasonic for 1 h. The supernatant was centrifugalized by 3000 rpm and finally collected by the centrifugation of 11000 rpm. Afterwards, the precipitate power was soaked in 3.0 mol L^{-1} HCl solution for removing impurities of metal oxide byproducts and then washed with DI water for several times until pH reaches ≈ 7, then centrifugated and dried at 60 °C for 12 h.

1.2.2 Synthesis of SiO_x/iron-nitrogen co-doped carbon (SiO_x/Fe-N-C)

First, 0.30 g polyacrylonitrile (PAN, MW=150,000) was completely dissolved into 10 mL N,N-dimethylformamide (DMF). Second, 0.196 g SiO_2 NPs were subsequently added into the above mixed solution of PAN/DMF by ultrasonic treatment for 30 min. Third, 0.118 g

$Fe(acac)_3$ were added, and stirred vigorously for 12 h under room temperature was directly added into the SiO_2/PAN/DMF solution and vigorously stirred overnight at room temperature. The mixed solution was obtained for the subsequent electrospray process with a 10 mL syringe. The receiving distance between the 22-gauge needle and aluminum collector was 15 cm. The electrospray process was conducted with a positive voltage of 15 kV at a flow rate of 0.6 mL h^{-1}. Finally, the $Fe(acac)_3$/SiO_2/PAN powder was directly calcinated at 230 ^oC with a heating rate of 1 ^oC min^{-1} for 2 h in air and then subsequently carbonized at 650 ^oC with a heating rate of 2 ^oC min^{-1} for 2 h under N_2 atmosphere. The obtained material was designated as SiO_x/Fe-N-C. The products carbonized at different temperatures of 550 ^oC and 750 ^oC were denoted as C1 and C2. Moreover, SiO_x/N−C was prepared through similar processes without adding metal salts.

1.2.3 Synthesis of SiO_x/metal-nitrogen co-doped carbon (SiO_x/M-N-C, M=Co, Ni)

For the universal synthesis of SiO_x/Co-N-C and SiO_x/Ni-N-C, 0.118 g $Fe(acac)_3$ was replaced by 0.119 g $Co(acac)_3$ and 0.086 g $Ni(acac)_2$, respectively. The detailed electrospray and carbonization processes were the same as that of the SiO_x/Fe-N-C sample.

1.3 Characterization of as-synthesized materials

A series of the products were tested by X-ray diffraction (XRD) on a Bruker D8 Advanced X-ray Diffractometer (Cu-Kα radiation: λ=0.15406 nm) for the phase analysis. Scanning electron microscope (SEM, Zeiss_Supra55) was employed for studying the morphology of the samples at an acceleration voltage of 5.0 kV. High-resolution transmission electron microscopy (HRTEM) images and energy dispersive X-ray spectroscopy mapping images were captured on a Tecnai G2 F30 transmission electron microscopy at an acceleration

voltage of 300 kV. High-angle annular dark-field scanning transmission electron microscopy (HAADF-STEM) was performed on TEM Titan Themis. X-ray photoelectron spectroscopy (XPS) tests were conducted on a Thermo Scientific ESCALAB 250 apparatus. EXAFS measurements were performed at beamline BL14W1 at the Shanghai Synchrotron Radiation Facility. Additionally, Brunauer-Emmett-Teller (BET) and Barrett-Joyner-Halenda (BJH) tests were conducted on the Autosorb IQ3 instrument. Thermogravimetry analysis (TGA) was performed under O_2 atmosphere from room temperature to 800 ^{o}C at a heating rate of 10 ^{o}C min^{-1}.

1.4 Electrochemical measurements

Electrochemical measurements were carried out using 2032 coin-type cells. The anodes were prepared by mixing 70 wt% active materials, 20 wt% super P, 5 wt% carboxymethyl cellulose (CMC) binder, and 5 wt% butadiene styrene rubber (SBR) in deionized water, of which the slurry was coated on Cu foil and subsequently dried at 60 °C under vacuum for overnight. The anode and bare Li foil are utilized to assemble the Li-ion half-cell. 1 M $LiPF_6$ in ethylene carbonate (EC) and diethyl carbonate (DEC) (volume ratio of 1:1) with 5% FEC additive was employed as the electrolyte and Celgard 2400 film was used as a separator. Galvanostatic cycling tests were conducted at a potential range of 0.01-3.0 V (vs. Li^+/Li) based on the Land CT2001A system. Besides, the cyclic voltammetry (CV) curves and the electrochemical impedance spectra (EIS) were conducted on a CHI 660E instrument.

1.5 Computational details.

All spin-polarized DFT calculations for periodic material systems were performed with the Vienna Ab initio simulation package (VASP)[1] with the projector-augmented wave (PAW)

method[2]. The exchange–correlation function was handled using the generalized gradient approximation (GGA) formulated by the Perdew-Burke-Ernzerhof (PBE). The van der Waals (vdW) interactions are described with the DFT-D3 method in Grimme's scheme[3]. The interaction between the atomic core and electrons was described by the projector augmented wave method. The plane-wave basis set energy cutoff was set to 500 eV. The Brillouin zone was sampled with Gamma (Γ) centered Monkhorst-Pack mesh sampling (3 × 3 × 1 for SiO_x/N-C and SiO_x/Fe-N-C, 3 × 3 × 3 for α-SiO_2) for geometry relaxation and 11 × 11 × 1 for high-quality density of states (DOS) calculations. All the slabbed models possessed five atomic layers and a vacuum spacing of ≈15 A˚ sampled, ensuring negligible lateral interaction of adsorbates. The bottom two layers were kept frozen at the lattice position. All structures with a dynamic magnetic moment were fully relaxed to optimize without any restriction until their total energies were converged to $< 1\times10^{-5}$ eV, and the average residual forces were < 0.02 eV/Å[4]. For an accurate electrostatic potential, the DFT calculation was modified with the dipole correction[5] in the Z-direction.

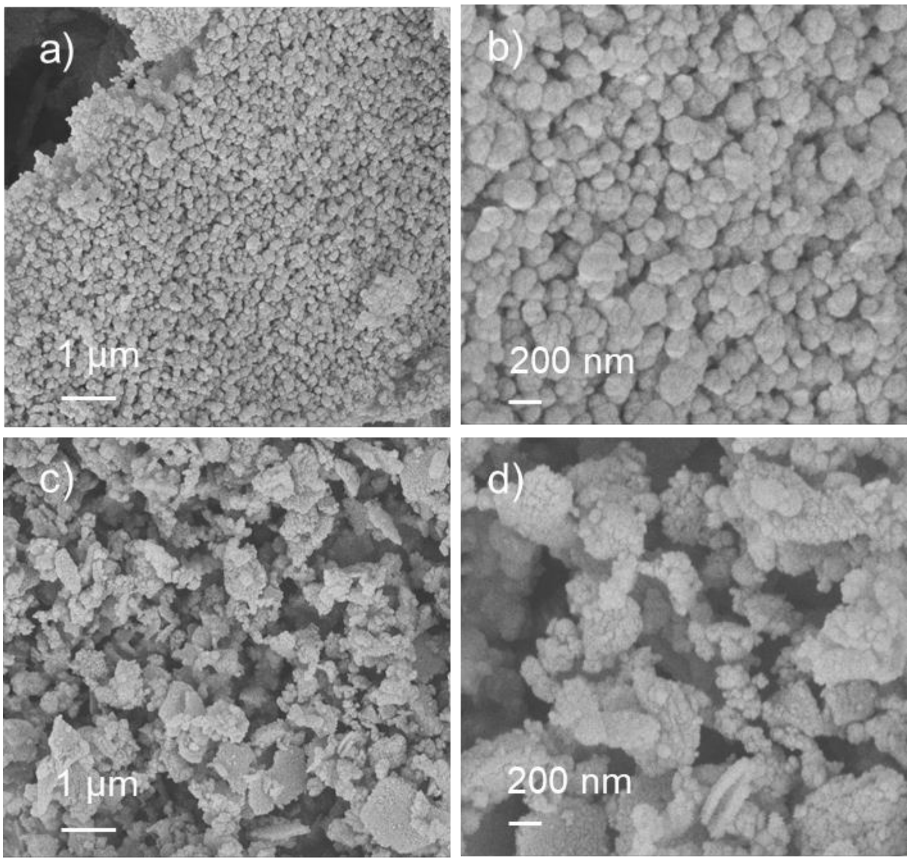

Figure S1. (a,b) SEM images of SiO_2 powder directly obtained from the calcination of RHs; (c,d) SEM images of SiO_2 NPs pretreated by the ultrasonic and centrifugal processes.

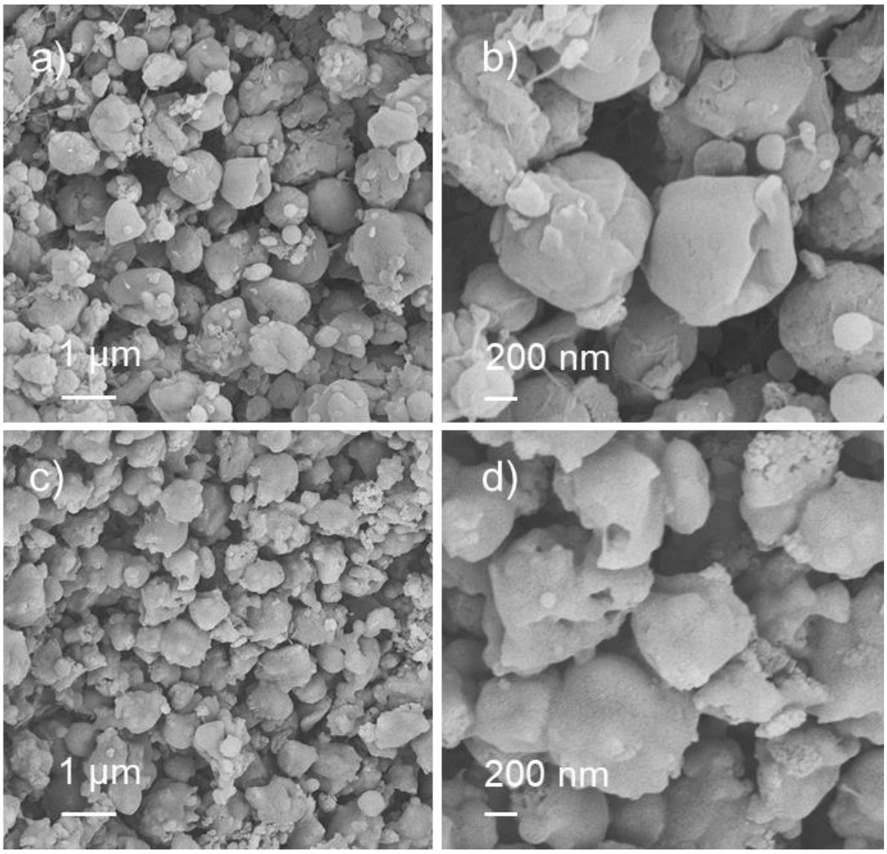

Figure S2. (a,b) SEM images of SiO_2/PAN; (c,d) SEM images of SiO_x/N-C.

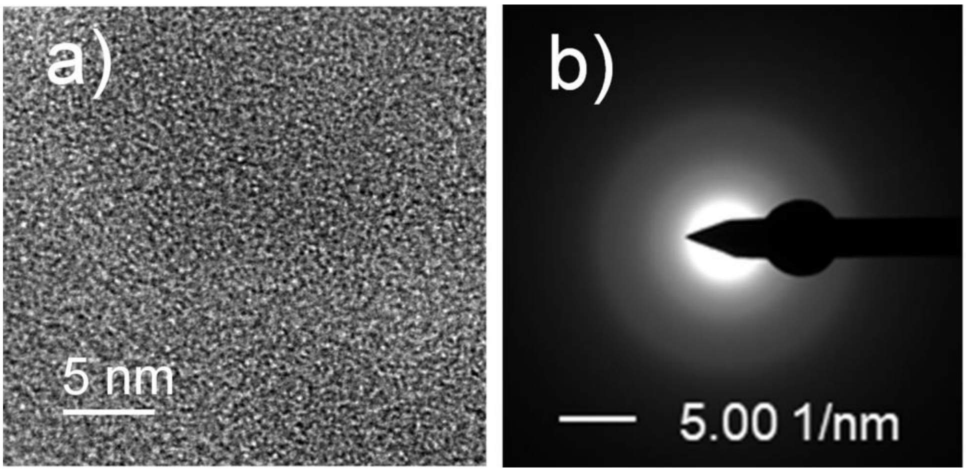

Figure S3. (a) HRTEM image of SiO_x/Fe-N-C; (b) SAED pattern of SiO_x/Fe-N-C.

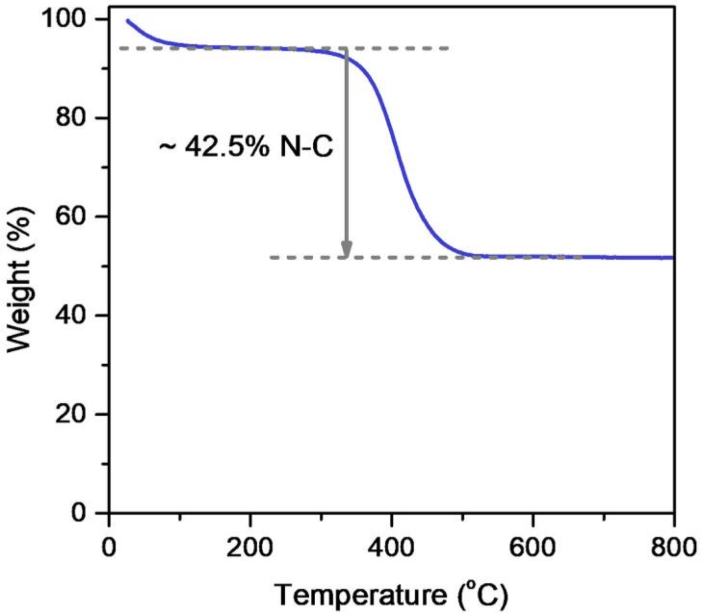

Figure S4. TG curve of the SiO_x/Fe-N-C sample under O_2 temperature.

As shown in **Figure S4**, the weight content of N-C in SiO_x/Fe-N-C is ~ 42.5%. Therefore, the weight content of the remaining SiO_x and atomically dispersed Fe are determined to be 57.5%.

The mass of atomically dispersed Fe should be 0.0187 g based on the addition of 0.118 g $Fe(acac)_3$ into the precursor solution. ((0.118 g/353.17 g mol^{-1})*55.85 g mol^{-1}=0.0187 g).

As shown in **Figure S8**b, the weight contents of Si^{2+}, Si^{3+} and Si^{4+} in the Si2p spectrum are 24.98%, 44.34% and 30.68%. The average valence of Si is calculated to be 3.06. Compared to the valence of Si in SiO_2, the mass of SiO_x should be 3.06/4*0.196 g=0.1499 g.

Comparing the mass of SiO_x (0.1499 g) and Fe (0.0187 g), the mass ratios of SiO_x and Fe species are 88.91% and 11.09%. Thus, the weight content of SiO_x and Fe species should be 51.5% and 6.4%. (88.91%*57.5% ≈ 51.1%; 11.09%*57.5% ≈ 6.4%).

Based on the above calculations, the mass ratios of atomically dispersed Fe, SiO_x and N-C are 6.4%, 51.1% and 42.5%, respectively.

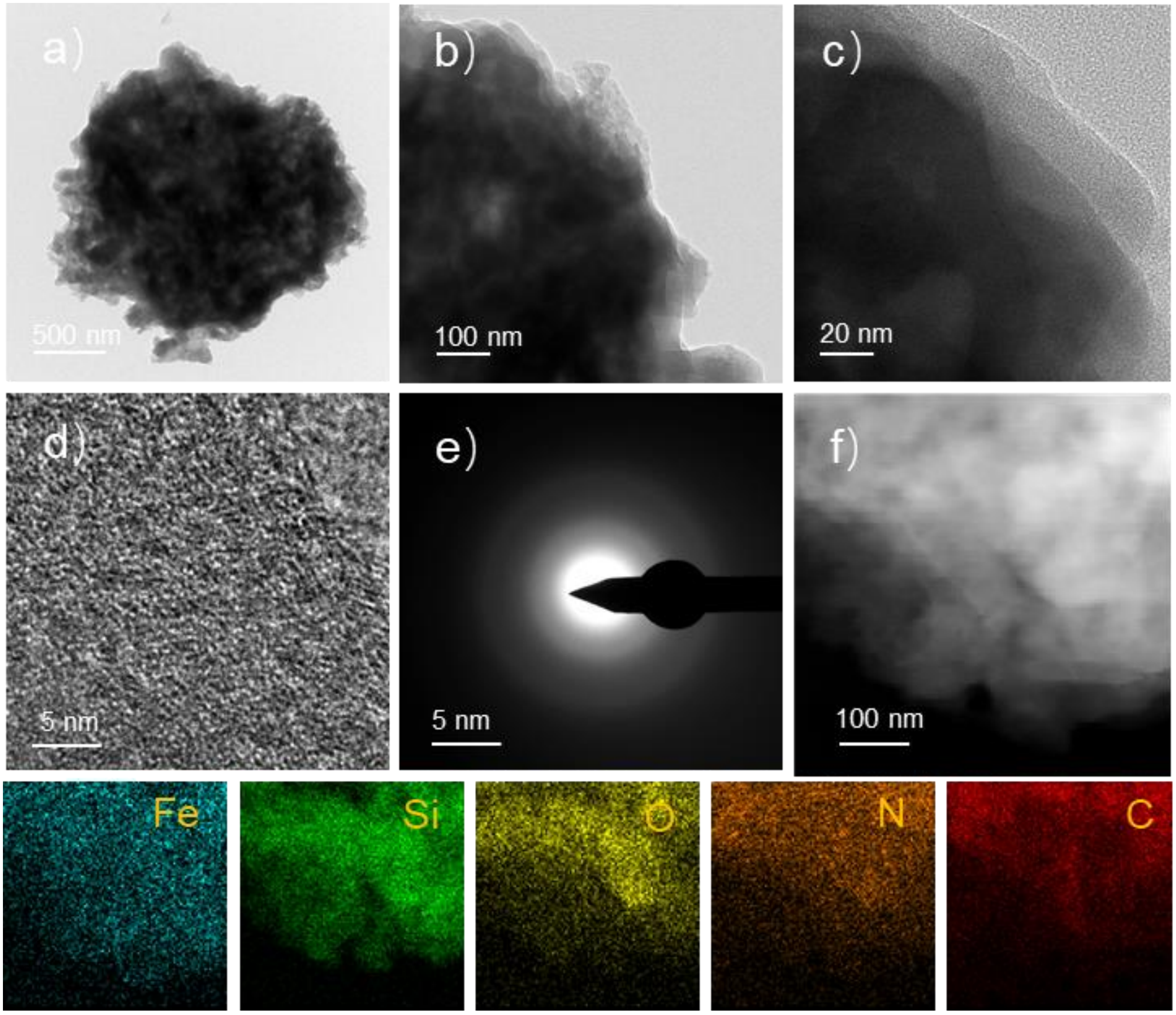

Figure S5. (a-c) TEM images of C1 at different magnifications; (d,e) HRTEM and SAED image of C1; (f) EDS mapping results of C1.

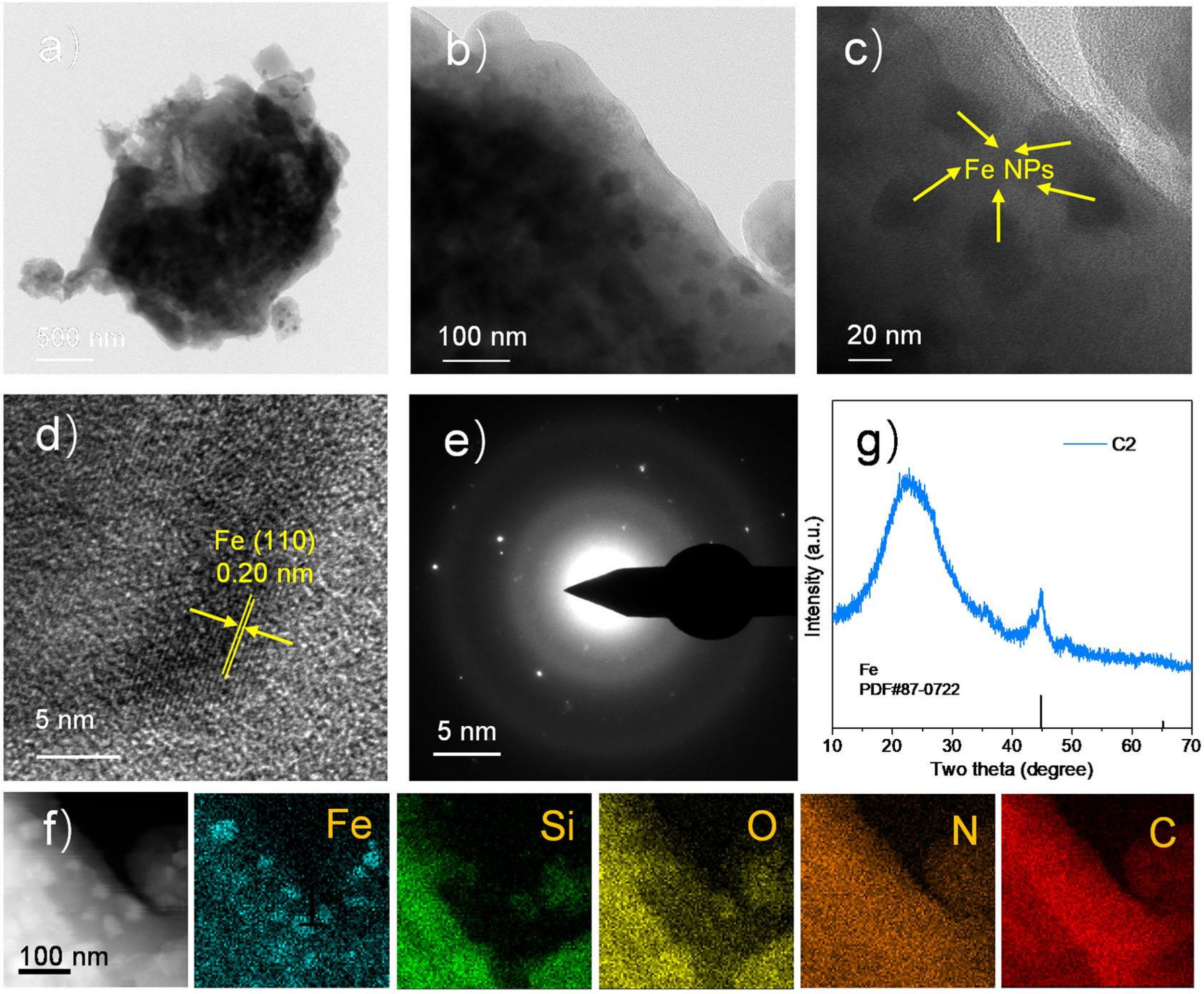

Figure S6. (a-c) TEM images of C2 at different magnifications; (d, e) HRTEM and SAED images of C2; (f) EDS mapping results of C2; (g) XRD curve of C2.

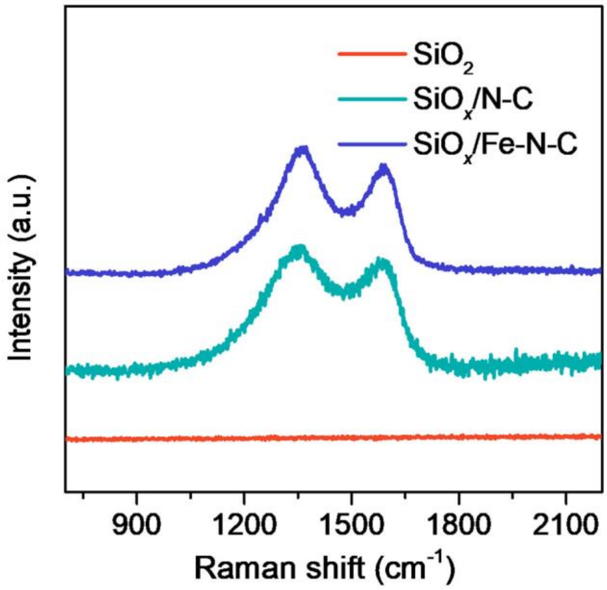

Figure S7. Raman patterns of pure SiO_2, SiO_x/N-C and SiO_x/Fe-N-C.

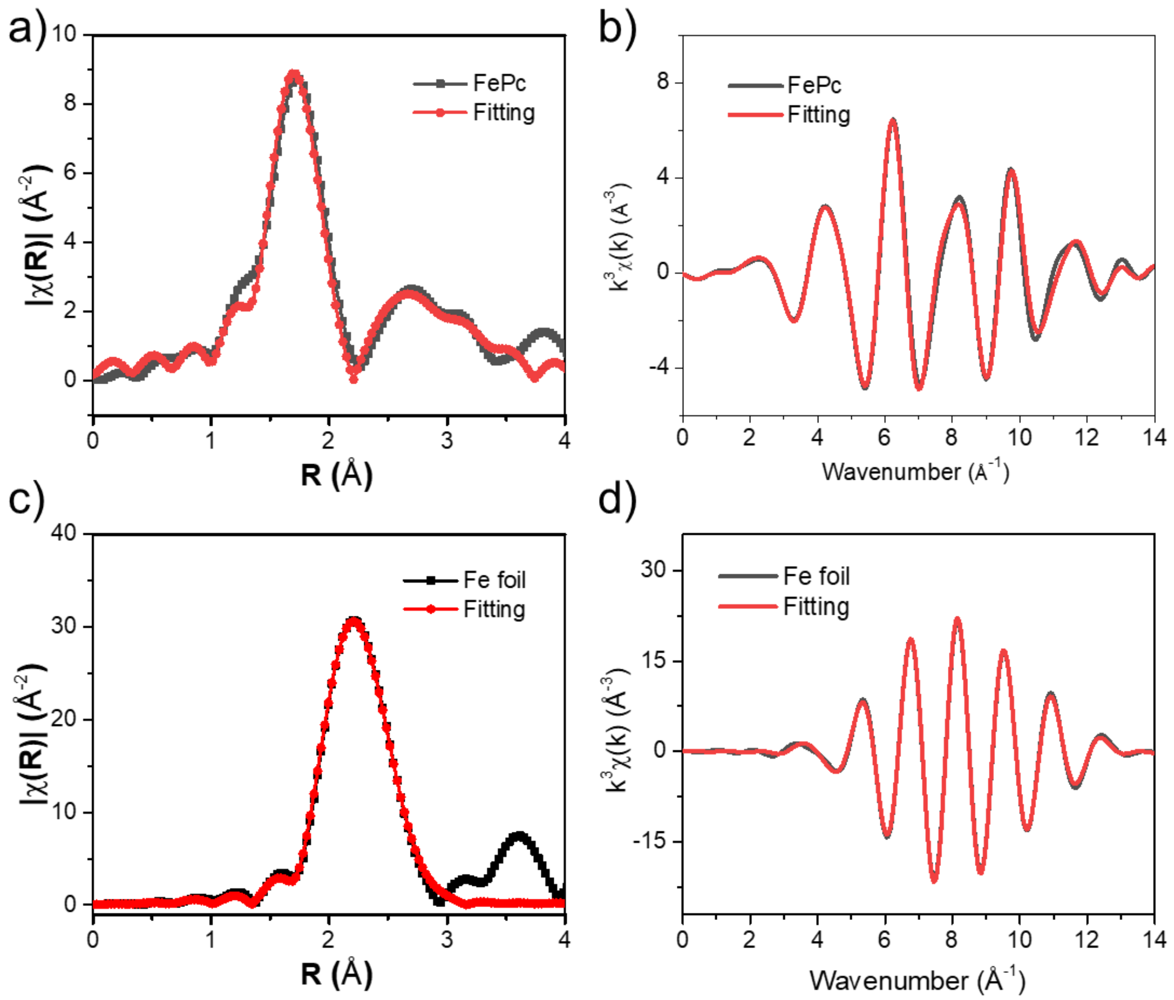

Figure S8. The corresponding EXAFS K-space and R-space fitting curves of (a, b) FePc; (c, d) Fe foil.

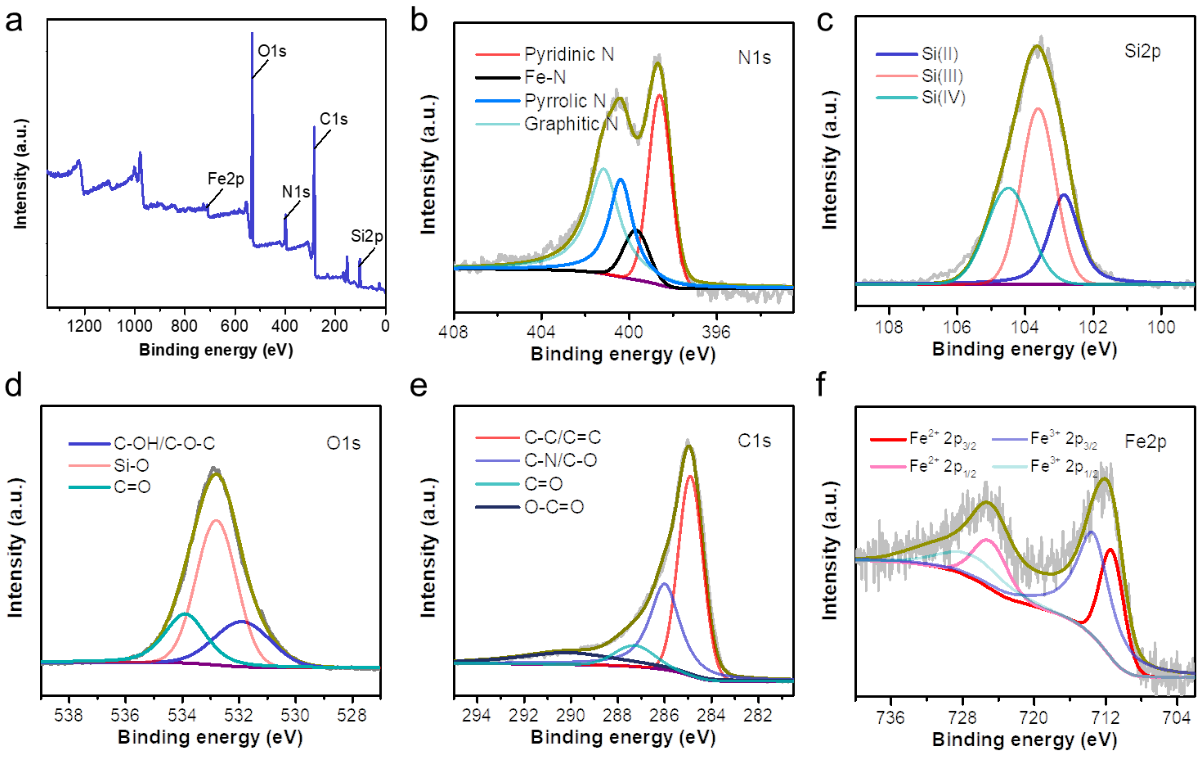

Figure S9. XPS characterization of SiO_x/Fe-N-C. (a) Full XPS spectrum; (b-f) XPS high-resolution spectra of N 1s, Si 2p, O 1s, C 1s and Fe 2p.

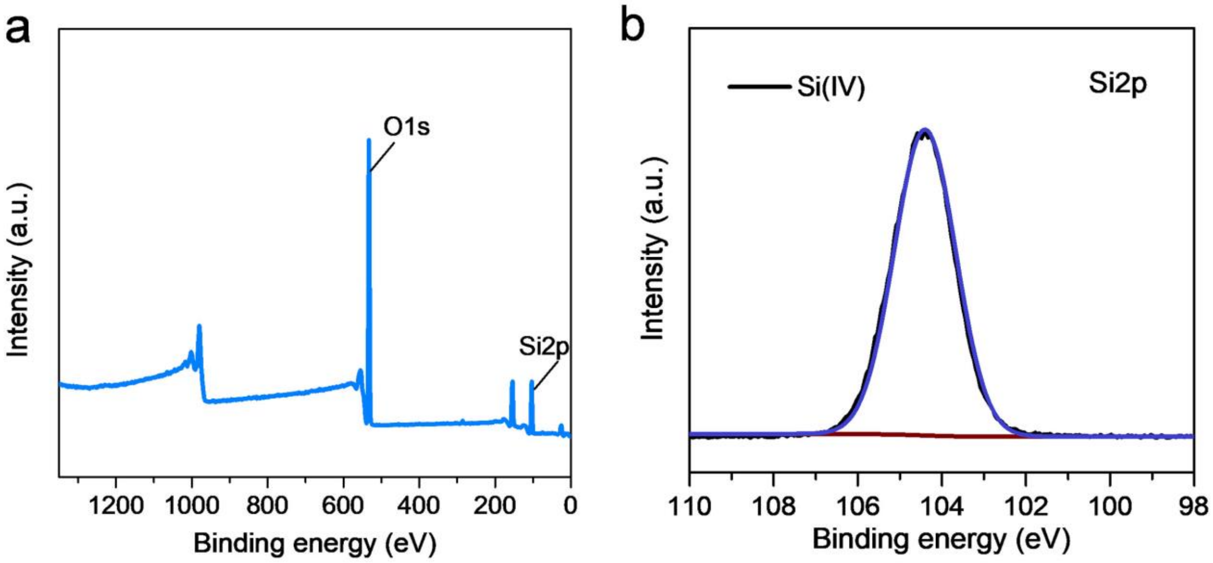

Figure S10. (a) Full XPS spectrum of SiO_2; (b) Si 2p spectrum of SiO_2.

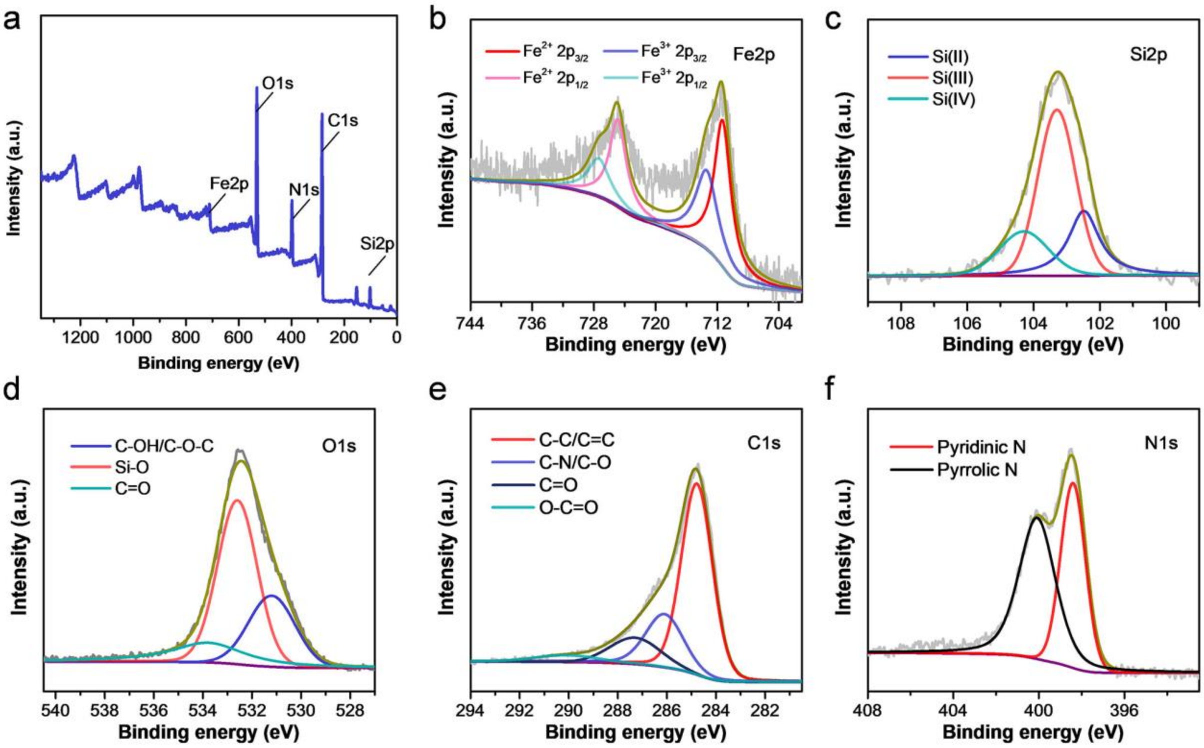

Figure S11. XPS characterization of C1. (a) Full XPS spectrum; (b-f) XPS high-resolution spectra of Fe 2p, Si 2p, O 1s, C 1s and N 1s.

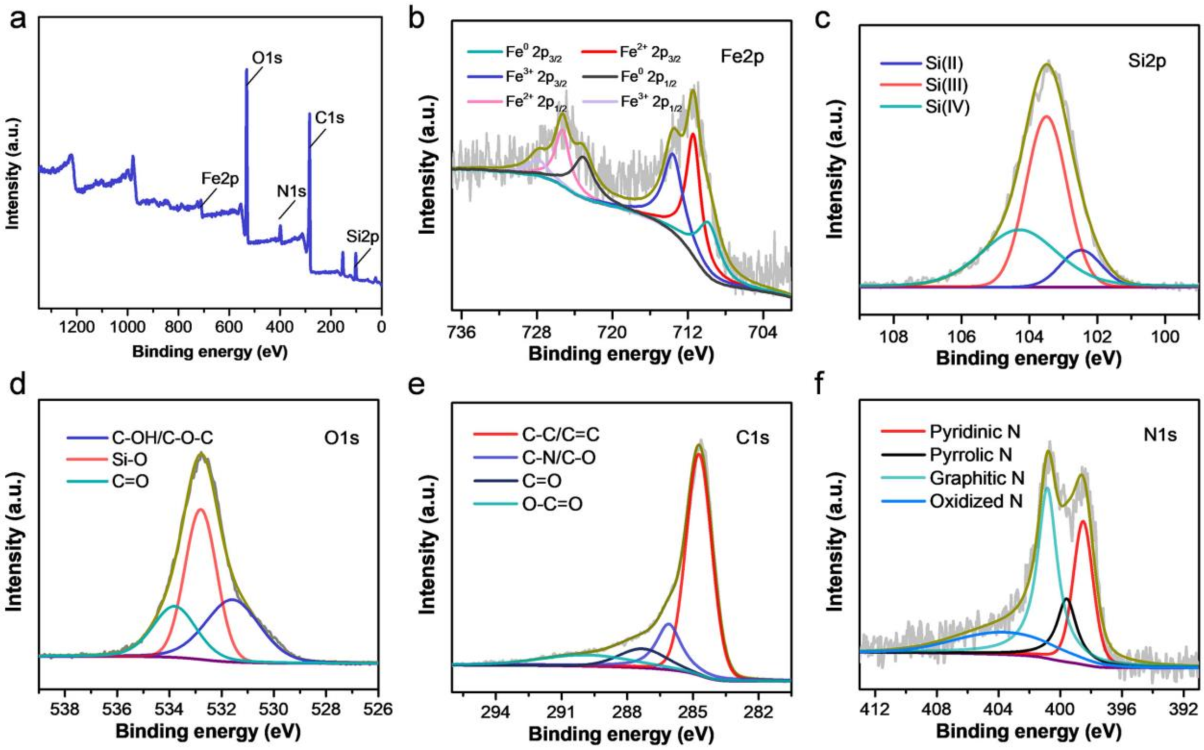

Figure S12. XPS characterization of C2. (a) Full XPS spectrum; (b-f) XPS high-resolution spectra of Fe 2p, Si 2p, O 1s, C 1s and N 1s.

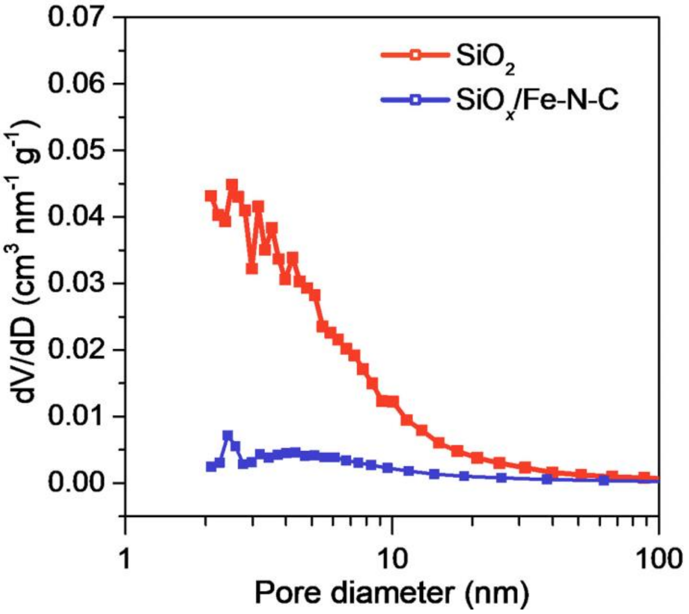

Figure S13. Pore size distributions of pure SiO_2 and SiO_x/Fe-N-C.

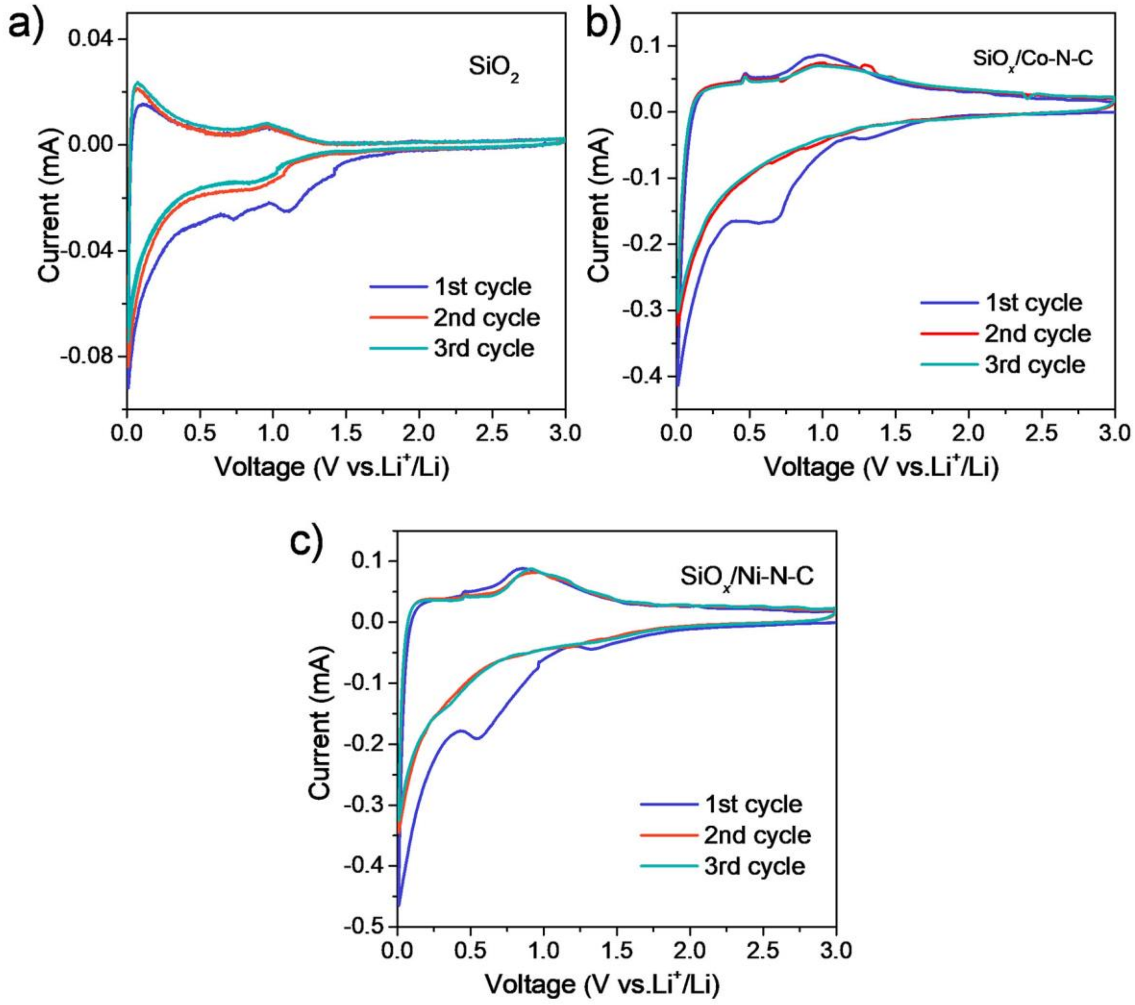

Figure S14. CV curves of (a) pure SiO_2; (b) SiO_x/Co-N-C; (c) SiO_x/Ni-N-C.

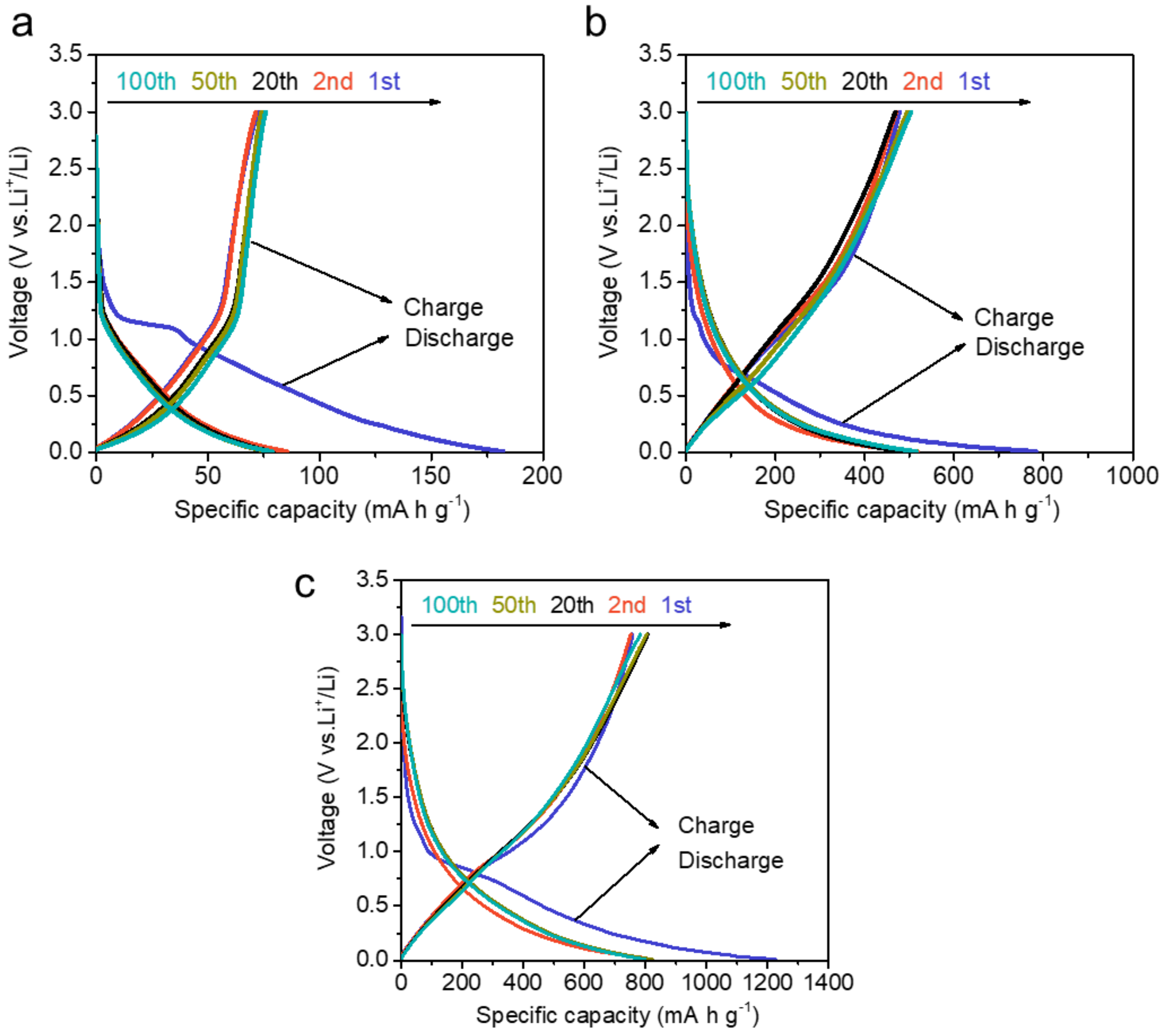

Figure S15. GCD curves at 100 mA g^{-1}. (a) Pure SiO_2; (b) SiO_x/N-C; (c) SiO_x/Fe-N-C.

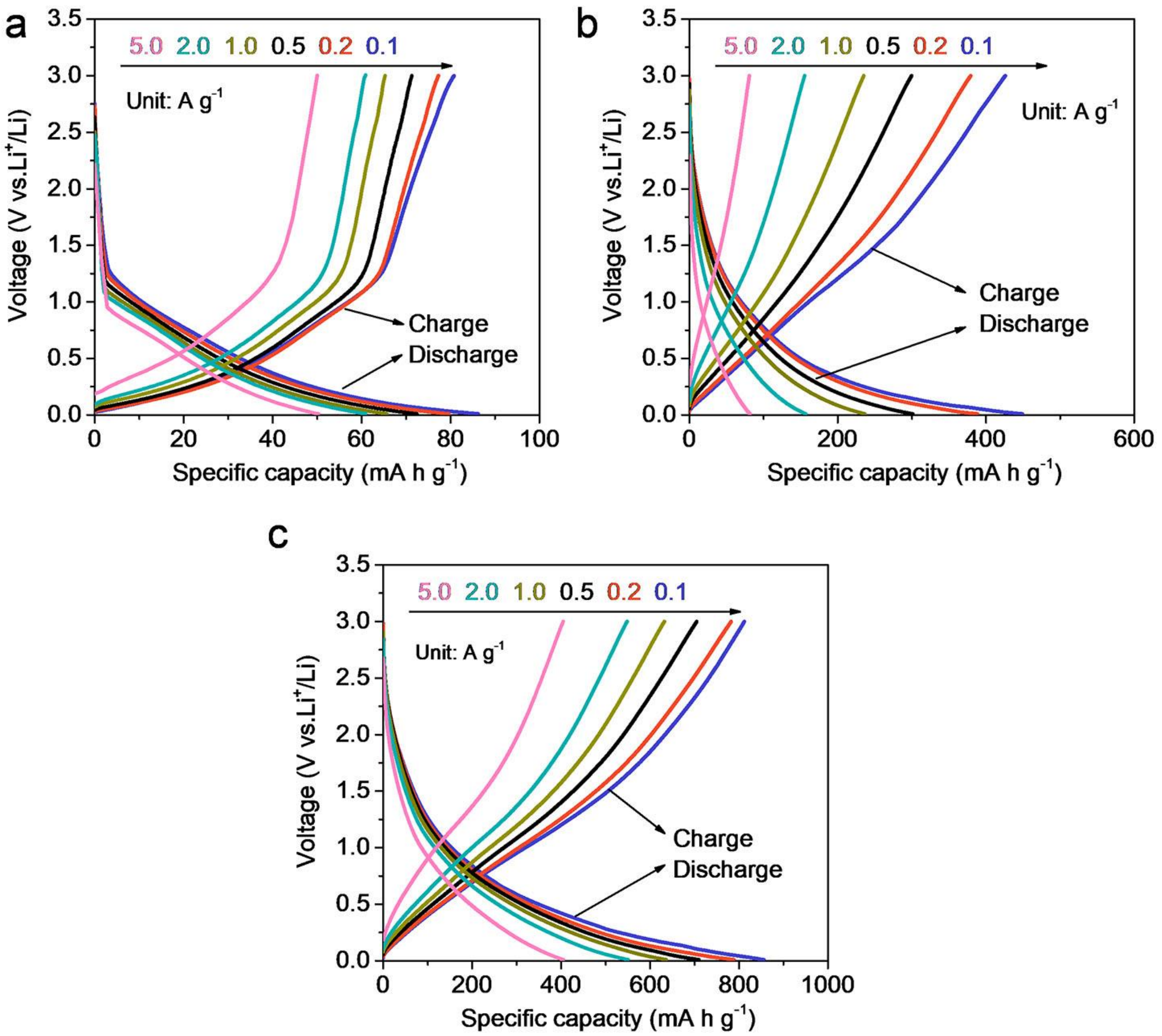

Figure S16. GCD curves at various current densities. (a) Pure SiO_2; (b) SiO_x/N-C; (c) SiO_x/Fe-N-C.

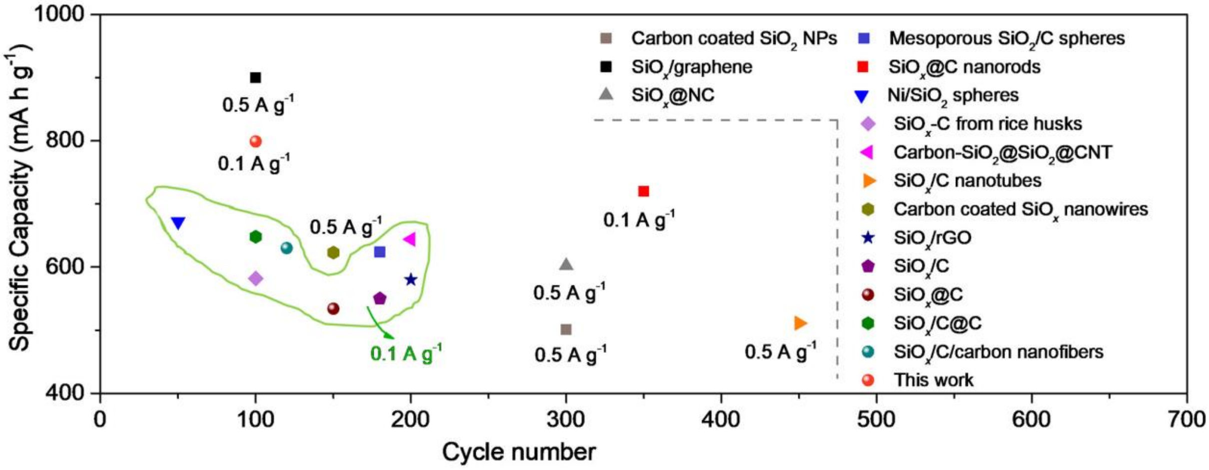

Figure S17. Comparison of the cycle stability with the ever-reported SiO_x-based anodes, which is obtained according to **Tables S3.**

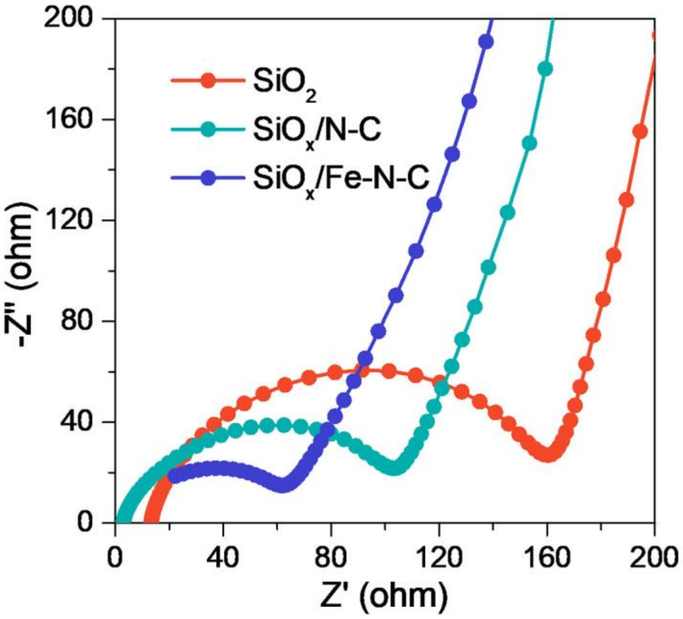

Figure S18. Nyquist plots of the SiO_2, SiO_x/N-C and SiO_x/Fe-N-C samples before cycling.

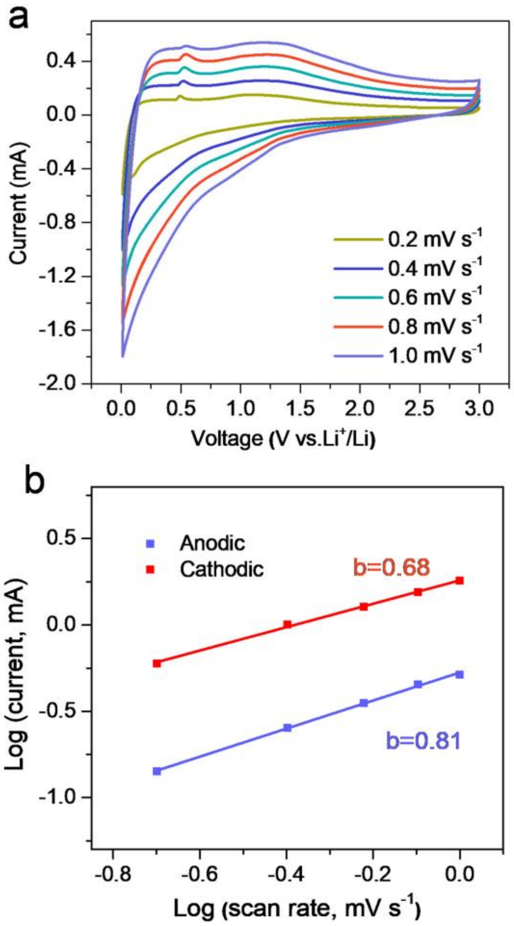

Figure S19. (a) CV curves of the SiO_x/Fe-N-C anode at various sweep rates including 0.2, 0.4, 0.6, 0.8 and 1.0 mV s^{-1}; (b) Relationship of the SiO_x/Fe-N-C anode between the peak currents (i) and scan rates (*v)*.

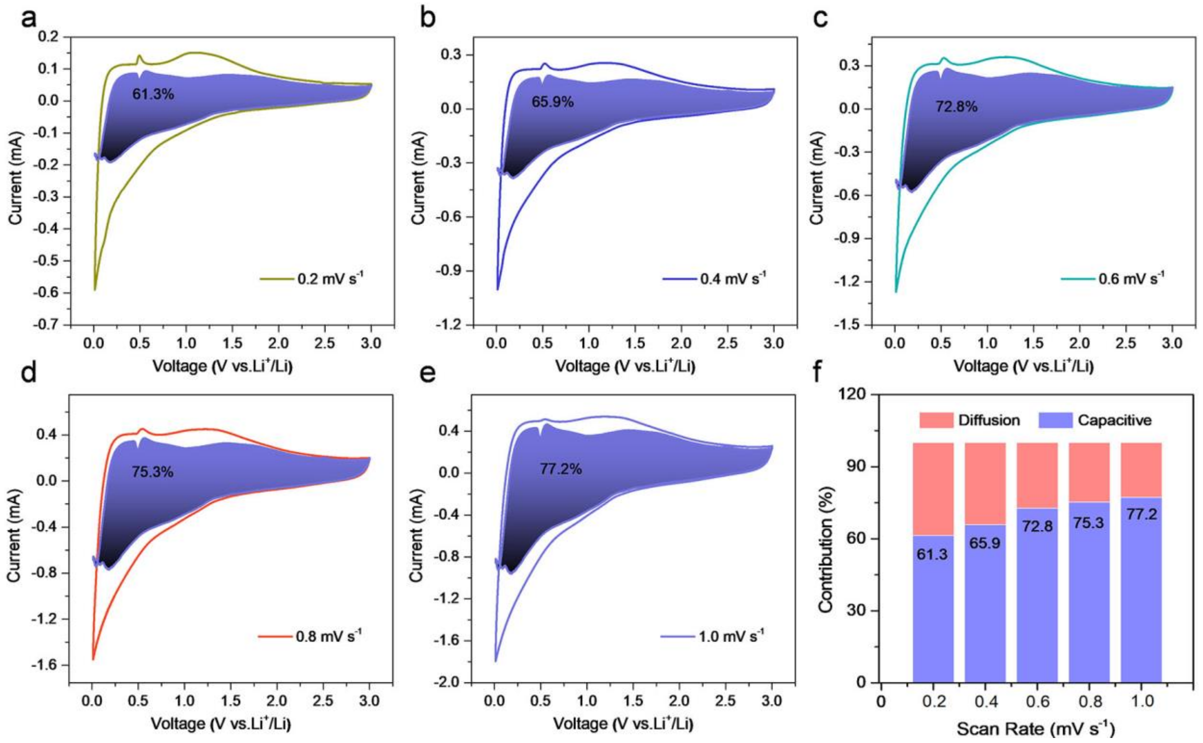

Figure S20. (a-e) Pseudocapacitive contributions of the SiO_x/Fe-N-C anode at various scan rates; (f) Bar chart of the pseudocapacitive and diffusion contributions of the SiO_x/Fe-N-C anode.

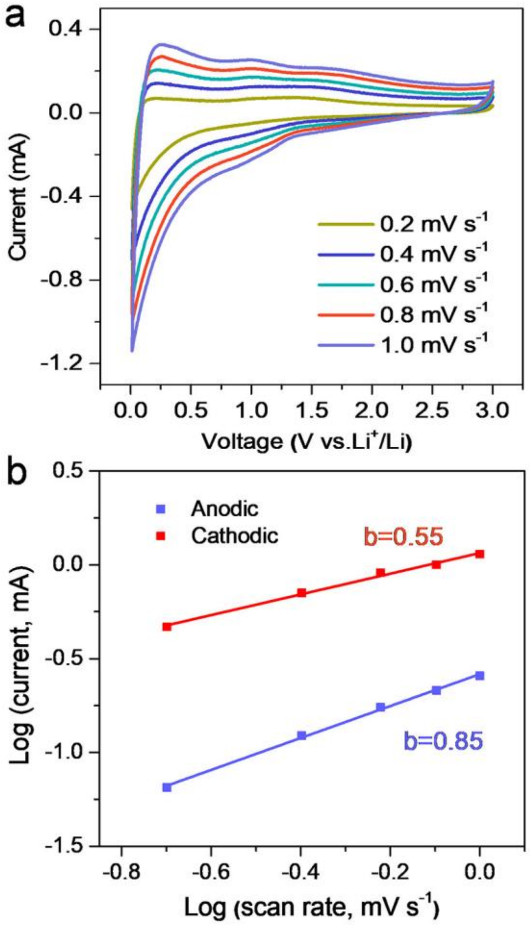

Figure S21. (a) CV curves of the SiO_x/N-C anode at various sweep rates including 0.2, 0.4, 0.6, 0.8 and 1.0 mV s^{-1}; (b) Relationship of the SiO_x/N-C anode between the peak currents (i) and scan rates (*v*).

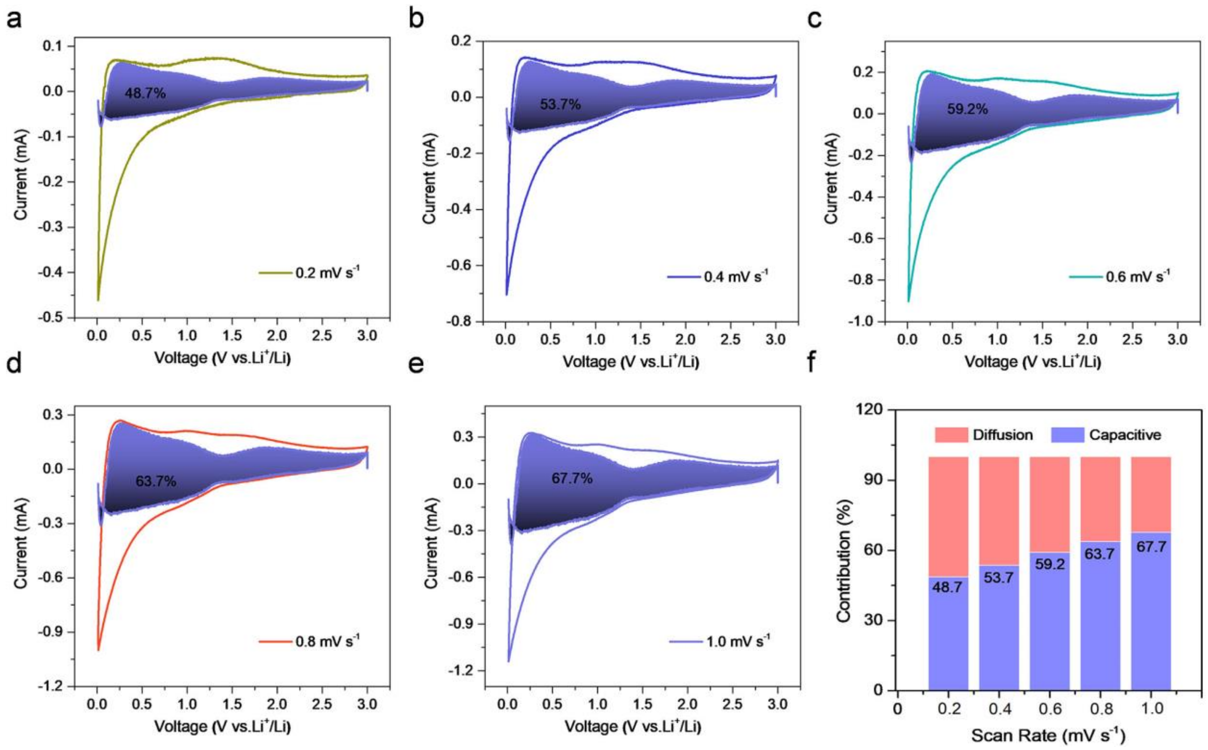

Figure S22. (a-e) Pseudocapacitive contributions of the SiO_x/N-C anode at various scan rates; (f) Bar chart of the pseudocapacitive and diffusion contributions of the SiO_x/N-C anode.

Table S1. The weight content of C, N, Fe, and O by XPS, TG, and ICP-OES.

Sample	Si(%)	O(%)	C(%)	N(%)	Fe(%)
TG	51.1 (Calculated SiO_x)		42.5 (N-C)		6.4 (Calculated)
XPS	16.8	34.1	34.5	8.4	6.2
ICP-OES	—	—	—	—	6.1

Table S2. EXAFS fitting parameters at the Fe K-edge.

Sample	Path	C.N.	R (Å)	σ^2*10^3 (Å^2)	ΔE (eV)	R_{factor}
Fe foil	Fe-Fe	8*	2.46±0.01	4.83±1.33	5.32±2.18	0.0056
	Fe-Fe	6*	2.84±0.02	5.68±2.30	5.05±3.54	
SiO_x/Fe-N-C	Fe-N	4.8	1.92±0.02	8.73±1.03	-7.74±5.13	0.0021
	Fe-C	6.0	2.93±0.11	9.81±3.99		
FePc	Fe-N	5.1	2.12±0.19	1.85±0.53	8.54±2.56	0.0080
	Fe-C	7.0	3.14±0.18	1.27±1.14		

Illustration: C.N.: coordination number; R: bond distance; σ^2, Debye–Waller factors; ΔE: the inner potential correction; *R* factor: the goodness of the fit; *: the experimental EXAFS fit of metal foil by fixing C.N. as the known crystallographic value.

Table S3. Comparison of the lithium-storage performance between the SiO_x/Fe-N-C sample and similar materials reported recently.

Materials	Content of SiO_2 or SiO_x (wt%)	1st DC/CC (mA h g^{-1})/ICE(%)	Capacity (mA h g^{-1})	Cycles	Current density (mA g^{-1})
Carbon coated SiO_2 NPs[6]	__	1380/765/55.4	501	300	500
Mesoporous SiO_2/C spheres[7]	94	__	624	180	100
SiO_x/graphene[8]	63.5	> 1000/-/69	> 900	100	500
SiO_x@C nanorods[9]	65.4	1324/906/68.4	~720	350	100
SiO_x@NC[10]	59	__	602	300	500
Ni/SiO_2 hierarchical hollow Spheres[11]	40.65	1195/676/56.6	672	50	100
SiO_x-C from rice husks[12]	__	998.5/466/46.67	582.1	100	100
Carbon-SiO_2@SiO_2@CNT[13]	~72.1	__	644	200	100
SiO_x/C nanotubes[14]	__	1425/921/64.6	511	450	500
Carbon coated SiO_x nanowires[15]	~90.67	2215/-/-	623	150	500
SiO_x/rGO[16]	__	__	580	200	100
SiO_x/C[17]	__	887.2/507.4/57.19	550	180	100
SiO_x@C[18]	65.5	1195/759/63.5	534	150	100
SiO_x/C@C[19]	72.22	1443/-/54.4	648	100	100
SiO_x/C/carbon nanofibers[20]	61.67	1283/1061/79.5	630	120	100
SiO_x/Fe-N-C **(This work)**	~51	1226.9/758.3/61.8	799.1	100	100

ICE: Initial Coulombic efficiency

Table S4. Pseudocapacitive contributions of the SiO_x/N-C and SiO_x/Fe-N-C anodes at various scan rates.

Sample	0.2 mV s^{-1}	0.4 mV s^{-1}	0.6 mV s^{-1}	0.8 mV s^{-1}	1.0 mV s^{-1}
SiO_x/N-C	48.7%	53.7%	59.2%	63.7%	67.7%
SiO_x/Fe-N-C	61.3%	65.9%	72.8%	75.3%	77.2%

Reference

[1] G. Kresse, J. Furthmüller, *Comput. Mater. Sci.* **1996**, *6*, 15.

[2] P. E. Blöchl, *Phys. Rev. B* **1994**, *50*, 17953.

[3] S. Grimme, J. Antony, S. Ehrlich, H. Krieg, *J. Chem. Phys.* **2010**, *132*, 154104.

[4] Z. F. Huang, J. Song, Y. Du, S. Xi, S. Dou, J. M. V. Nsanzimana, C. Wang, Z. J. Xu, X. Wang, *Nat. Energy* **2019**, *4*, 329.

[5] J. Neugebauer, M. Scheffler, *Phys. Rev. B* **1992**, *46*, 16067.

[6] G. Zhu, F. Zhang, X. Li, W. Luo, L. Li, H. Zhang, L. Wang, Y. Wang, W. Jiang, H. K. Liu, S. X. Dou, J. Yang, *Angew. Chem. Int. Ed.* **2019**, *58*, 6669.

[7] C. W. Wang, K. W. Liu, W. F. Chen, J. De Zhou, H. P. Lin, C. H. Hsu, P. L. Kuo, *Inorg. Chem. Front.* **2016**, *3*, 1398.

[8] Q. Xu, J. K. Sun, Z. L. Yu, Y. X. Yin, S. Xin, S. H. Yu, Y. G. Guo, *Adv. Mater.* **2018**, *30*, 1707430.

[9] Y. Ren, M. Li, *J. Power Sources* **2016**, *306*, 459.

[10] D. Liu, Z. Han, J. Ma, L. Gao, J. Cai, L. Zhang, S. Cheng, J. Xie, *Chem. Eng. J.* **2021**, *420*, 129754.

[11] C. Tang, Y. Liu, C. Xu, J. Zhu, X. Wei, L. Zhou, L. He, W. Yang, L. Mai, *Adv. Funct. Mater.* **2018**, *28*, 1704561.

[12] Y. Ju, J. A. Tang, K. Zhu, Y. Meng, C. Wang, G. Chen, Y. Wei, Y. Gao, *Electrochim.*

Acta **2016**, *191*, 411.

[13] L. Wang, X. Zhu, K. Tu, D. Liu, H. Tang, J. Li, X. Li, Z. Xie, D. Qu, *Electrochim. Acta* **2020**, *354*, 136726.

[14] Z. Wang, L. Kong, Z. Guo, X. Zhang, X. Wang, X. Zhang, *Chem. Eng. J.* **2022**, *428*, 131060.

[15] Z. Li, Q. He, L. He, P. Hu, W. Li, H. Yan, X. Peng, C. Huang, L. Mai, *J. Mater. Chem. A* **2017**, *5*, 4183.

[16] D. Liu, C. Chen, Y. Hu, J. Wu, D. Zheng, Z. zhong Xie, G. Wang, D. Qu, J. Li, D. Qu, *Electrochim. Acta* **2018**, *273*, 26.

[17] B. Zhang, H. Wang, C. Liu, D. Li, H. K. Kim, C. Harris, C. yen Lao, A. Abdelkader, K. Xi, *J. Alloys Compd.* **2019**, *801*, 658.

[18] Z. Wang, N. Yang, L. Ren, X. Wang, X. Zhang, *Microporous Mesoporous Mater.* **2020**, *307*, 110480.

[19] Y. Zhang, G. Hu, Q. Yu, Z. Liu, C. Yu, L. Wu, L. Zhou, L. Mai, *Mater. Chem. Front.* **2020**, *4*, 1656.

[20] Y. Zheng, X. Kong, I. Usman, X. Xie, S. Liang, G. Cao, A. Pan, *Inorg. Chem. Front.* **2020**, *7*, 1762.
